# DSPLMF: A Method for Cancer Drug Sensitivity Prediction Using a Novel Regularization Approach in Logistic Matrix Factorization

**DOI:** 10.3389/fgene.2020.00075

**Published:** 2020-02-27

**Authors:** Akram Emdadi, Changiz Eslahchi

**Affiliations:** ^1^ Department of Computer Sciences, Faculty of Mathematics, Shahid Beheshti University, Tehran, Iran; ^2^ School of Biological Sciences, Institute for Research in Fundamental Sciences (IPM), Tehran, Iran

**Keywords:** cancer, drug response, recommender system, matrix factorization, personalized treatment

## Abstract

The ability to predict the drug response for cancer disease based on genomics information is an essential problem in modern oncology, leading to personalized treatment. By predicting accurate anticancer responses, oncologists achieve a complete understanding of the effective treatment for each patient. In this paper, we present DSPLMF (**D**rug **S**ensitivity **P**rediction using **L**ogistic **M**atrix **F**actorization) approach based on Recommender Systems. DSPLMF focuses on discovering effective features of cell lines and drugs for computing the probability of the cell lines are sensitive to drugs by logistic matrix factorization approach. Since similar cell lines and similar drugs may have similar drug responses and incorporating similarities between cell lines and drugs can potentially improve the drug response prediction, gene expression profile, copy number alteration, and single-nucleotide mutation information are used for cell line similarity and chemical structures of drugs are used for drug similarity. Evaluation of the proposed method on CCLE and GDSC datasets and comparison with some of the state-of-the-art methods indicates that the result of DSPLMF is significantly more accurate and more efficient than these methods. To demonstrate the ability of the proposed method, the obtained latent vectors are used to identify subtypes of cancer of the cell line and the predicted IC50 values are used to depict drug-pathway associations. The source code of DSPLMF method is available in https://github.com/emdadi/DSPLMF.

## Introduction

Cancer is a genetic disease that results when cellular changes and accumulation of different types of mutations cause the uncontrolled growth and division of cells. There are more than 200 different types of cancer, having a significant global impact on public health. Since cancer is a disease of genetic complexity and diversity, the drug response for different patients can be different. The main reason for this occurrence is the difference in the molecular and genetic information of individuals, such as gene expression data, the type of mutation in the genome and copy number alteration information. These findings and achievements have recently made a significant challenge in the prediction of drug response for an individual patient in the research of precision medicine.

High-throughput drug screening technologies on several panels of cancer cell lines have been provided. For instance, two recent consortiums Genomics of Drug Sensitivity in Cancer (GDSC) Yang et al. (2012) and Cancer Cell Line Encyclopedia (CCLE) Barretina et al. (2012) have collected around 1,000 cell lines and their pharmacological profiles for several cancer drugs. The IC50 measure (minimal concentration of drug that induced 50% cell line death) is usually used as a sensitivity measure. To facilitate and speed up drug discovery and prediction process, many methods have been developed in these fields by researches from numerous domains such as computational biology, machine learning, and data mining approaches.

In the challenge of the DREAM project, the performance of 44 drug response prediction algorithms was considered for breast cancer cell lines. The introduced algorithms were evaluated using the weighted probabilistic c-index (WPC-index) and resampled Spearman correlation Costello et al. (2014). Various machine learning methods have been proposed in this area. Barretina et al. proposed a method for predicting drug response based on naive Bayes classifier that selected importance features by two steps. First, they used Wilcoxon Sum Rank Test and Fisher Exact Test to select the 30 top features and then they applied naive Bayes classifier for drug response prediction Barretina et al. (2012). SVM-RFE method is a wrapper that used SVM classifier and recursive feature selection method Dong et al. (2015). FSelector method used *k*-nearest neighbor (KNN) algorithm based on selected features that are achieved by information entropy Soufan et al. (2015). Suphavilai et al. (2018) proposed the CaDRReS method as a predictor cancer drug response model based on the recommender system and learning projections for drugs and cell lines into a latent space. AutoBorutaRF was presented by Xu et al., based on feature selection for classification of anticancer drug responses. The method first built a subset of essential features, then used Boruta algorithms Kursa et al. (2010) to select some features for applying Random-Forest classifier to predict drug response Lu et al. (2019).

In this paper, we modeled the cancer drug sensitivity problem based on “Recommender Systems” approach. A logistic matrix factorization algorithm was used for predicting drug cancer response. By applying the proposed model to GDSC and CCLE datasets, we proved that DSPLMF is of excellent prediction accuracy.

## Materials and Method

### Datasets

The performance of drug response prediction algorithms was evaluated on two benchmark datasets, including GDSC and CCLE. The datasets were downloaded by using R package PharmacoGx Smirnov et al. (2015). In these datasets, there are several types of information such as IC50 values according to the set of cell lines and drugs and some other information such as gene expression profile, copy number alteration, and single-nucleotide mutation that used in the model designing for more efficiency. Since in these datasets some of the above information is missing, the method of compensating for missing values given by Lu et al. (2019) is used. The missing value for a cell line can belong to response value, copy number alteration, and single-nucleotide mutation features. The cell lines with more than 50% missing value were removed from the dataset and for remaining, the missing values were predicted from the known values of *k*-nearest cell lines. At the end, 555 cell lines and 98 drugs remain without any missing value for GDSC and 363 cell lines and 24 drugs for CCLE datasets.

### Method

The main idea of the model DSPLMF is to construct a classification model for predicting how a cell line responds to a drug. Since drug response can be divided into two classes “sensitivity” and “resistance,” there are many ways for the purpose of classification based on IC50 values. By considering the histograms of IC50, we observed some histograms are normal-like, and others have skewness. Also, it can be supposed that the labels of classes should be determined by the data of individual drugs. For normal-like histograms, median, and mean are the same. If the histogram is skewed right, the mean is greater than the median, and if the histogram is skewed left, the mean is smaller than the median. We chose medium because we wanted to set a single, universal standard threshold for all drugs. So, the strategy introduced by Li et al. (2015) was used and the median of IC50 values were applied as a threshold for classification. The "sensitivity" or class with label 1 was assigned to a cell line if its IC50 is smaller than the median of cell lines for an individual drug and "resistance" or class with label 0 to a cell line was assigned, otherwise. DSPLMF method has four main steps as follows.

**Figure 1:**
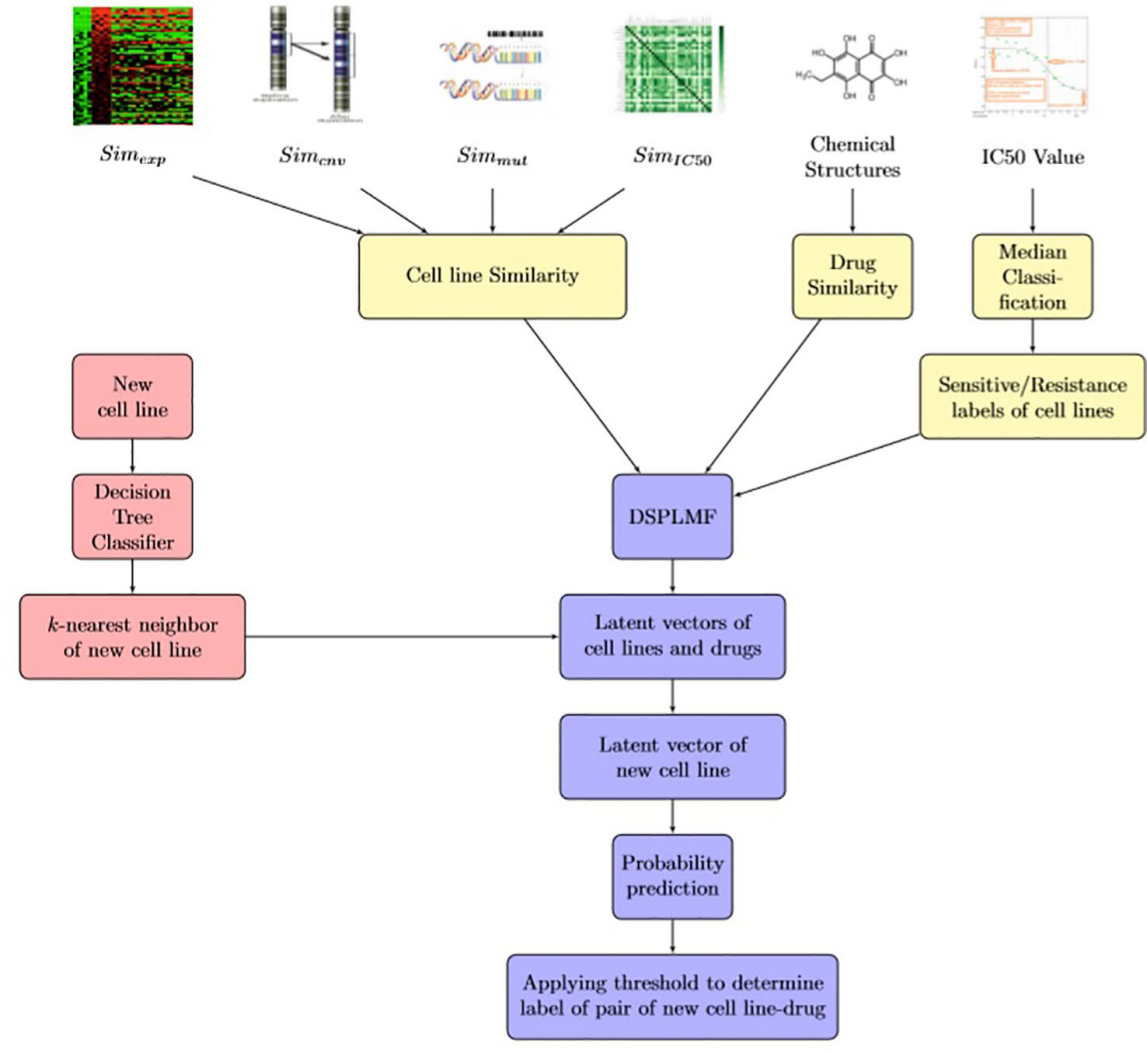
Scheme of DSPLMF algorithm. First, similarities between each pair of cell lines are constructed based on the information of gene expression, single-nucleotide mutation, copy number alteration, and IC50 values. Also, similarity between each pair of drugs is defined based on chemical substructure and the median of IC50 values are applied as a threshold for classification. Using DSPLMF model, the latent vectors for each cell line and drug are achieved. For each new cell line, decision tree classifier is applied to find its *t*-most nearest neighbors and the probabilities that this cell line is sensitive to drugs are estimated based on the latent vectors of its neighbors. Eventually, a threshold is applied on probabilities to assign sensitive or resistance class to each new cell line-drug pair.

In the first step, by converting the model to a classification problem, a 0,1 observation matrix was achieved, as cell lines and drugs are rows and columns of the matrix, respectively. Then, a logistic matrix factorization method for constructing the latent vectors for each cell line and drug is applied. In the second step, for improving the prediction accuracy of the model, the similarity information for cell lines and drugs are used. In the third step, a model is applied to learn to predict the probability that a new cell line would sensitive to a drug. Subsequently, with applying the threshold to predicted probabilities of the cell line-drug pairs, we classified each pair to sensitive or resistance class. In the next section, first the similarity matrices used in the model, were introduced and then the details of each step are explained in the following steps. The main scheme of DSPLMF algorithm is represented in **Figure 1**.

#### Similarity Matrix

##### Cell Line Similarity

In this part, the four similarities between each pair of cell lines based on the information of gene expression, single-nucleotide mutation, copy number alteration, and IC50 values were defined.

- **Gene expression Similarity,** Sim_exp_ Gene expression information is an auxiliary feature for similarity between cell lines. Let *e* *_i_* denoted the gene expression vector of cell line *c_i_* in cancerous conditions. For pair of cell lines *c_i_* and *c_j_*, *Sim_exp_*(*c_i_*, *c_j_*) is defined as the Pearson correlation between the vectors *e_i_* and *e_j_* and the gene expression similarity matrix between cell lines considered as *Sim_exp_* = [*Sim_exp_*(*c_i_*, *c_j_*)]*_n_*_×_ *_n_*, where n is the numbers of cell lines. Each entry of these metrics is in [−1,1]. The numbers of considered genes for two datasets GDSC and CCLE for similarity measure are 11,712 and 19,389, respectively. So the length of vector *e_i_* is 11,712 and 19,389 for GDSC and CCLE dataset, respectively.Q[SpecialChar] Verify that all the equations and special characters are displayed correctly.
- **Single-nucleotide mutation Similarity**, Sim_mut_ Let zero-one vectors *m_i_* indicate that whether a mutation occurred in the set of genes for cell line *c_i_* or not. *Sim_mut_*(*c_i_*, *c_j_*) is defined as the Jaccard similarity between the vectors *m_j_* and *m_j_* and the single-nucleotide mutation similarity matrix between cell lines considered as *Sim_mut_* = [*Sim_mut_*(*c_i_*, *c_j_*)]*_n_*_×_ *_n_*. Each entry of these metrics is in [0, 1]. The mutation information of 54 genes are accessible for cell lines in GDSC dataset and 1667 genes for cell lines in CCLE dataset, respectively.
- **Copy number alteration Similarity,** Sim_cnv_ Let *v_i_* denoted the copy number alteration vector for cell line *c_i_*. *Sim_cnv_*(*c_i_*, *c_j_*) is defined as the Pearson correlation between the vectors *v_i_* and *v_j_* and the copy number alteration similarity matrix between cell lines considered as *Sim_cnv_* = [*Sim_cnv_*(*c_i_*, *c_j_*)]*_n_*_×_ *_n_*. Each entry of these metrics is in [−1, 1]. The information of copy number alteration of 24,959 and 24,960 genes for two GDSC and CCLE datasets are accessible, respectively.
- **IC50 value Similarity,** Sim_IC50_ Moreover, the similarity between cell lines proposed by Liu et al. (2018) based on the correlation between their response IC50 values was used. Let *IC_i_* denoted the vector of IC50 values of drugs in cell line *c_i_*. *Sim_IC_*_50_(*c_i_*, *c_j_*) is defined as the Pearson correlation between the vectors *IC_i_* and *IC_j_* and the similarity based on IC50 matrix between cell lines considered as *Sim_IC_*_50_ = [*Sim_IC_*_50_(*c_i_*, *c_j_*)]*_n_*_×_ *_n_* and each element of these metrics in [−1, 1].

To aggregate these similarities to a single matrix, *Sim_total_* = [*SC_ij_*]*_n_*_×_ *_n_*, the following formula is used:

(1) $${\mathrm{Sim}}_{total}=\frac{\lambda Sim_{exp}+\gamma Sim_{cnv}+\phi Sim_{mut}+\psi Sim_{IC50}}{\lambda +\gamma +\phi +\psi }$$

where γ, *λ*, *ϕ* and *ψ* are parameters that represent the importance of each of the matrix and tuned in the model. The numbers of considered genes for two datasets GDSC and CCLE for *Sim_exp_* are 11,712 and 19,389, respectively. The mutation information of 54 genes is accessible for cell lines in GDSC dataset and 1,667 genes for cell lines in CCLE *dataset*. The information of copy number alteration of 24,959 and 24,960 genes for two GDSC and CCLE datasets are accessible, respectively. Since three matrices *Sim_exp_*, *Sim_cnv_*, and *Sim_mut_* have been constructed by different sets of genes (the number of common genes between them is about 50%), there is not an additive relation between them. In general, an absolute correlation coefficient of >0.7 among two or more predictors indicates the presence of collinearity. But as **Table 1** shows, all correlation coefficients between similarity matrices are very low, so there is not collinearity between matrices and they can be linearly combined.

**Table 1:** Correlation coefficient between four matrices *Sim_exp_*, *Sim_cnv_*, *Sim_mut_*, and *Sim_IC50_*.

Correlation Coefficient	*Sim_exp_*	*Sim_cnv_*	*Sim_mut_*	*Sim_IC50_*
*Sim_exp_*	1.0	0.24	−0.11	0.19
*Sim_cnv_*	0.24	1.0	0.14	0.015
*Sim_mut_*	−0.11	0.14	1.0	−0.06
*Sim_IC50_*	0.19	0.015	−0.06	1.0

##### Drug Similarity, Sim_drug_

Since it is expected that similar drugs have the same effect on cell lines, drug similarity information for predicting drug response was used in the proposed method. A drug can be represented as a binary feature vector, by using drug substructures, drug transporters, drug targets, drug enzymes, drug pathways, drug indications, or drug side effects information. Since there is only information about chemical substructures, for each drug we have a zero-one vector of size 881, where 881 is the number of known chemical substructures of a drug. In this vector one indicates the presence of a substructure of drug and zero otherwise. We downloaded the substructure for each drug from PubChem. The PubChem system generates a binary substructure fingerprint for chemical structures. These fingerprints are used by PubChem for similarity neighboring and similarity searching. Let $V_{d_{i}}$ and $V_{d_{j}}$ are the vectors correspond to the drugs *d_i_* and *d_j_*. Similarity (*di*, *dj*) is considered as Jaccard similarity between these two vectors. We construct the matrix *Sim_drug_* = [*SD_ij_*]*_m_*_×_ *_m_* as similarity matrix between each pair of drugs.

#### Logistic Matrix Factorization

Assume the set of cell lines is denoted by C = {*c*_1_, *c*_2_, …, *c_n_* } and the set of drugs is denoted by D = {*d*_1_, *d*_2_, …, *d_m_* }, where n and m are the numbers of cell lines and the numbers of drugs, respectively. The relationship between cell lines and drugs are represented by a binary matrix *Q* = [*q_ij_*]*_n_*_×_ *_m_*, where each element *q_ij_* ∈ {0, 1}. If a cell line is *c_i_* sensitive to a drug *d_j_*, *q_ij_* = 1 and otherwise *q_ij_* = 0. The probability of sensitivity of a cell line to a drug is defined by a logistic function as follows:

(2) $$p_{ij}=\frac{\exp\;(u_{i}v_{j}^{T}+\beta_{i}^{c}+\beta_{j}^{d})}{1+\exp\;(u_{i}v_{j}^{T}+\beta_{i}^{c}+\beta_{j}^{d})}$$

where *u_i_* nd *v_j_* are the latent vectors of size *L* corresponding to i-th cell line and j-th drug, respectively and the latent vectors of all cell lines and all drugs are denoted by *U* and *V*, respectively. On the other hands, the non-negative values $\beta_{i}^{c}$ and $\beta_{j}^{d}$ are the bias parameters according to cell line i and drug j, respectively. Moreover, we denoted *β^c^* ∈ ℝ*^n^* ^× 1^ and *β^d^* ∈ ℝ*^m^* ^× 1^ as bias vectors for cell lines and drugs, respectively. Bias parameters are considered because some cell lines respond significantly to many drugs and there are cell lines that respond to few drugs. Similarly for some drugs, there are many cell lines that respond to them, and there are drugs that most cell lines do not respond to significantly. Thus, by applying these parameters, we try to reduce bias. The vectors $\beta^{c}=(\beta_{1}^{c},\ldots ,\beta_{n}^{c})$and $\beta^{d}=(\beta_{1}^{d},\ldots ,\beta_{m}^{d})$ considered as bias vector of the model.

In this model, all the data in the training set are assumed to be independent. So the probability that matrix *Q* occurred, considering the latent and bias vectors, can be computed as:

(3) $$\begin{array}{c} p(Q|U,V,\beta^{c},\beta^{d})\\ =\left(\underset{1\leq i\leq n,1\leq j\leq m,q_{ij}=1}{\prod }{\left[p_{ij}^{q_{ij}}{\left(1-p_{ij}\right)}^{\left(1-q_{ij}\right)}\right]}^{r}\right)\times \left(\underset{1\leq i\leq n,1\leq j\leq m,q_{ij}=0}{\prod }p_{ij}^{q_{ij}}{\left(1-p_{ij}\right)}^{\left(1-q_{ij}\right)}\right) \end{array}.$$

When *q_ij_* = 1 then both *r* (1 – *q_ij_*) and 1 – *q_ij_* are zero. Similarly, when *q_ij_* = 0, *rq_ij_* = *q_ij_* = 0. So, formula 3 is rewritten as follows:

(4) $$\begin{array}{c} p(Q|U,V,\beta^{c},\beta^{d})\\ =\left(\underset{1\leq i\leq n,1\leq j\leq m,q_{ij}=1}{\prod }p_{ij}^{rq_{ij}}{\left(1-p_{ij}\right)}^{\left(1-q_{ij}\right)}\right)\times \left(\underset{1\leq i\leq n,1\leq j\leq m,q_{ij}=0}{\prod }p_{ij}^{rq_{ij}}{\left(1-p_{ij}\right)}^{\left(1-q_{ij}\right)}\right) \end{array}.$$

Finally, the above probability is shown as follows:

(5) $$p(Q|U,V,\beta^{c},\beta^{d})=\prod_{i=1}^{n}\prod_{j=1}^{m}p_{ij}^{rq_{ij}}{\left(1-p_{ij}\right)}^{\left(1-q_{ij}\right)}.$$

Where (*r* ≧̸ 1) is used to control the importance levels of observed interactions. In some classification problems with two classes (0 and 1), lack of information make us to assign label zero to some objects. But, it may be that the real label of these objects are one. So, the members of class one are highly trusted, while some members assign to class zero because of lack of information. As an example, in drug-target prediction or drug-drug interaction prediction models, the observed interacting drug-target pairs or drug-drug pairs have been experimentally verified; thus, they are more trustworthy and important than the unknown pairs. Toward more accurate modeling for these prediction models, the authors can assign higher importance levels to the interaction pairs than unknown pairs. This importance weighting strategy (considering *r* > 1) has been demonstrated to be effective for personalized recommendations. On the other hand, in DSPLMF model, both classes (sensitivity and resistance) have the same importance and validity. So, we set *r* to be one.

We also deposited zero-mean spherical Gaussian priors on latent vectors of cell lines and drugs as:

(6) $$p(U|\sigma_{c}^{2})=\prod_{i=1}^{n}N(u_{i}|0,\sigma_{c}^{2}I)$$

(7) $$p(V|\sigma_{d}^{2})=\prod_{j=1}^{m}N(v_{j}|0,\sigma_{d}^{2}I)$$

where *I* denotes the identity matrix and $\sigma_{c}^{2}$ and $\sigma_{d}^{2}$ are parameters for controlling the variances of prior distributions of cell lines and drugs. Based on Bayesian theorem we have:

(8) $$p(M|Q)=\frac{p(Q|M)p\left(M\right)}{p\left(Q\right)}.$$

Since *U*, *V*, *β^c^*, *β^d^* are the parameters in the model *M*, Bayesian theorem is as follows:

(9) $$p(U,V,\beta^{c},\beta^{d}|Q)=\frac{p(Q|U,V,\beta^{c},\beta^{d})p(U|\sigma_{c}^{2})p(V|\sigma_{d}^{2})}{p\left(Q\right)}.$$

So we can conclude the following relation:

(10) $$p(U,V,\beta^{c},\beta^{d}|Q)\propto p(Q|U,V,\beta^{c},\beta^{d})p(U|\sigma_{c}^{2})p(V|\sigma_{d}^{2}).$$

According to the Bayesian theorem and equations 5, 6, and 7, the log of the posterior distribution is estimated as follows:

(11) $$\begin{array}{c} \log\;p(U,V,\beta^{c},\beta^{d}|Q,\sigma_{c}^{2},\sigma_{d}^{2})=\sum_{i=1}^{n}\sum_{j=1}^{m}[rq_{ij}(u_{i}v_{j}^{T}+\beta_{i}^{c}+\beta_{j}^{d})-\left(1+rq_{ij}-q_{ij}\right)\log\;(1+\exp\;(u_{i}v_{j}^{T}+\beta_{i}^{c}+\beta_{j}^{d}))]-\frac{\lambda_{c}}{2}\sum_{i=1}^{n}||u_{i}|{|}_{2}^{2}-\frac{\lambda_{d}}{2}\sum_{j=1}^{m}||v_{j}|{|}_{2}^{2}+T \end{array}.$$

In formula 11, regarding how Bayesian theorem is applied to classification problems, we could convert the direct proportional relation between the left hand side and the numerator of the fraction of equation 10 to equalized, by adding constant term *T* to the formula. Where *T* is independent of the model parameters Hand et al. (1999). $\lambda_{c}=\frac{1}{\sigma_{c}^{2}}$, $\lambda_{d}=\frac{1}{\sigma_{d}^{2}}$. The parameters of the model can be learned by maximizing the above formula, which is equivalent to minimizing the following objective function:

(12) $$\begin{array}{c} \underset{U,V,\beta^{c},\beta^{d}}{\min}\;\sum_{i=1}^{n}\sum_{j=1}^{m}[(1+rq_{ij}-q_{ij})\log\;(1+\exp\;(u_{i}v_{j}^{T}+\beta_{i}^{c}+\beta_{j}^{d}))-rq_{ij}(u_{i}v_{j}^{T}+\beta_{i}^{c}+\beta_{j}^{d})]+\frac{\lambda_{c}}{2}||U|{|}_{F}^{2}+\frac{\lambda_{d}}{2}||V|{|}_{F}^{2} \end{array}$$

where ‖·‖*_F_* denotes the Frobenius norm of matrix.

For regularization the objective function 12, for each cell line *c_i_*_,_ we choose the set *N_k_*(*c_i_*) that denotes the *k*-most similar cell lines to *c* (except *c_i_*) using *Sim_total_* matrix. We constructed adjacency matrix *A= [a_ij_]_nxn_* that represents cell line neighborhood information as follow:

(13) $$a_{ij}=\{\begin{array}{ll} SC_{ij} & \;\;c_{j}\in N_{k}(c_{i})\\ 0 & \;\;otherwise \end{array}.$$

*A* is an *n* × *n* matrix, which for the row corresponding to cell line *c_i_*, the entries of columns corresponding to the *k*-most similar cell lines of *c_i_* are obtained from their similarities, *Sim_total_* matrix, and the other elements of this row are zero.

Similarly, for a drug *d_i_*, the set *N_k_*(*di*) denotes the *k*-most similar drugs to *d_i_* (except *d_i_*) using *Sim_drug_* matrix. The adjacency matrix *B* to describe the drug neighborhood information is denoted by *B* = [*b_ij_*]*_m_*_×_ *_m_*, where;

(14) $$b_{ij}=\{\begin{array}{ll} SD_{ij} & \;\;d_{j}\in N_{k}(d_{i})\\ 0 & \;\;otherwise \end{array}.$$

*B* is an *m* × *m* matrix, which for the row corresponding to drug *d_i_*, the entries of columns corresponding to the *k*-most similar drugs of *d_i_* are obtained from their similarities, *Sim_drug_* matrix, and the other elements of this row are zero.

**Figure 2:**
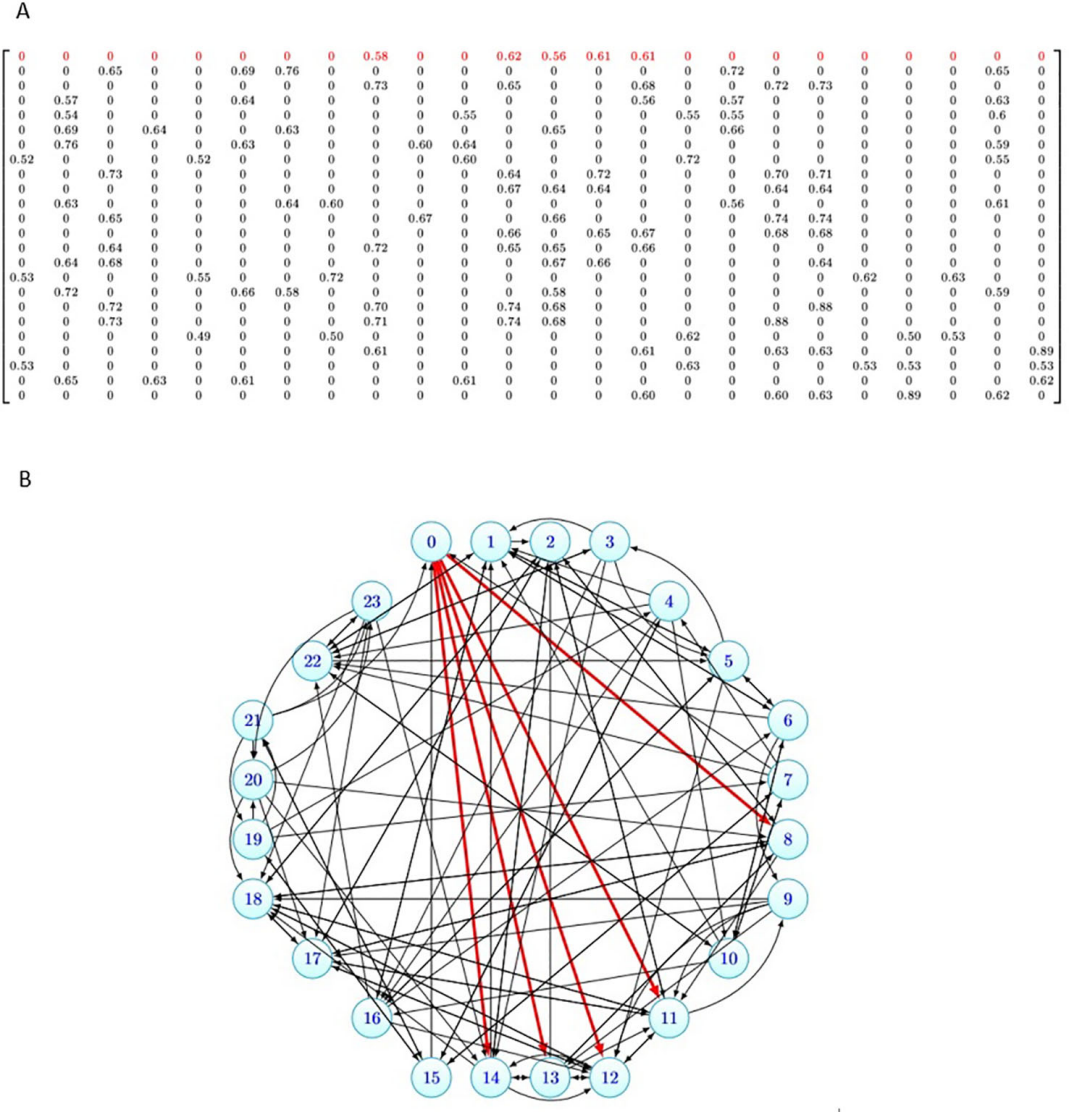
Data structure of the similarity matrix for 24 drugs in Cancer Cell Line Encyclopedia (CCLE) dataset. **(A)** The similarity matrix *B*_24 × 24_. **(B)** The graph corresponding to the similarity matrix *B*_24 × 24_.

To illustrate the data structure of these similarity matrices, as an example, for *k* = 5 and 24 drugs in CCLE dataset, the similarity matrix *B* is denoted in **Figure 2A**. **Figure 2B**, shows the graph corresponding to this matrix. As it can be seen from **Figure 2A**, each row *i* of the matrix has five nonzero elements corresponding to the five-most similar drugs of *d_i_* in *Sim_drug_* matrix, and the other elements are zero. In **Figure 2B**, the degree of each node is five and the red edges denote the neighbors of the nutlin-3. 5-most similar drugs to Nutlin-3 based on sim drug matrix are AEW541, AZD0530, Lapatinib, crizotinib, and sorafenib.

To minimize the distance between feature vector corresponding to cell line *i* and vectors of its nearest neighbors in latent space, we minimize two objective functions in formulas 15, 16 as follows:

(15) $$\begin{array}{c} \frac{\alpha }{2}\sum_{i=1}^{n}\sum_{j=1}^{n}(a_{ij}||u_{i}-u_{j}|{|}_{F}^{2})\\ =\frac{\alpha }{2}[\sum_{i=1}^{n}(\sum_{j=1}^{n}a_{ij})u_{i}u_{i}^{T}+\sum_{j=1}^{n}(\sum_{i=1}^{n}a_{ij})u_{j}u_{j}^{T}]-\frac{\alpha }{2}tr(U^{T}AU)-\frac{\alpha }{2}tr(U^{T}A^{T}U)=\frac{\alpha }{2}tr(U^{T}H^{c}U) \end{array}$$

(16) $$\begin{array}{c} \frac{\beta }{2}\sum_{i=1}^{m}\sum_{j=1}^{m}(b_{ij}||v_{i}-v_{j}|{|}_{F}^{2})\\ =\frac{\beta }{2}[\sum_{i=1}^{m}(\sum_{j=1}^{m}b_{ij})v_{i}v_{i}^{T}+\sum_{j=1}^{m}(\sum_{i=1}^{m}b_{ij})v_{j}v_{j}^{T}]-\frac{\beta }{2}tr\left(V^{T}BV\right)-\frac{\beta }{2}tr\left(V^{T}B^{T}V\right)=\frac{\beta }{2}tr\left(V^{T}H^{d}V\right) \end{array}$$

Where tr(.) is the trace of a matrix, $H^{c}=(E^{c}+{\tilde{E}}^{c})-(A+A^{T})$, *E^c^* and ${\tilde{E}}^{c}$ are two diagonal matrices and their diagonal elements are $E_{ii}^{c}=\sum_{j=1}^{n}\left(a_{ij}\right)$ and $\tilde{E_{\mathrm{jj}}^{c}}=\Sigma_{i=1}^{n}(a_{ij})$, $H^{d}=(E^{d}+{\tilde{E}}^{d})-(B+B^{T})$. *E^d^* and ${\tilde{E}}^{d}$ are two diagonal matrices and their diagonal elements are $E_{ii}^{d}=\sum_{j=1}^{m}\left(b_{ij}\right)$ and $\tilde{E_{\mathrm{jj}}^{d}}=\sum_{i=1}^{m}\left(b_{ij}\right)$. *α* and *β* are two parameters for weighting the similarity between cell lines and drugs, respectively.

The values of two matrices *A* and *B* show the similarity of the cell lines to each other and the similarity of the drugs to each other, respectively. Using the calculation of Frobenius norm multiplied by the elements in A and B is because of we would like more similar cell lines (drugs), have closer latent vectors in the latent space. But, the parameters α and β determine the effectiveness of these two matrices *A* and *B* in the objective function. By these strategies and tuning the parameters α and β, we determine the impact of cell line similarity and drug similarity in DSPLMF method.

By plugging two equations 15, 16 into formula 12, we will have the following:

(17) $$\begin{array}{c} \underset{U,V,\beta^{c},\beta^{d}}{\min}\;\sum_{i=1}^{n}\sum_{j=1}^{m}(1+rq_{ij}-q_{ij})\log\;(1+\exp\;(u_{i}v_{j}^{T}+\beta_{i}^{c}+\beta_{j}^{d}))-rq_{ij}(u_{i}v_{j}^{T}+\beta_{i}^{c}+\beta_{j}^{d})+\frac{\lambda_{c}}{2}||U|{|}_{F}^{2}+\frac{\alpha }{2}tr(U^{T}H^{c}U)+\frac{\lambda_{d}}{2}||V|{|}_{F}^{2}+\frac{\beta }{2}tr(V^{T}H^{d}V) \end{array}$$

Finally, we upgrade the formula 17 as follows:

(18) $$\begin{array}{c} \underset{U,V,\beta^{c},\beta^{d}}{\min}\;\sum_{i=1}^{n}\sum_{j=1}^{m}(1+rq_{ij}-q_{ij})\log\;(1+\exp\;(u_{i}v_{j}^{T}+\beta_{i}^{c}+\beta_{j}^{d}))-rq_{ij}(u_{i}v_{j}^{T}+\beta_{i}^{c}+\beta_{j}^{d})+\frac{1}{2}tr[U^{T}(\lambda_{c}I+\alpha H^{c})U]+\frac{1}{2}tr[V^{T}(\lambda_{d}I+\beta H^{d})V] \end{array}.$$

By this function, we try to predict the latent vectors of cell lines and drugs, where the similar cell lines or drugs have closer latent vectors to their KNNs.

For optimization the above function, the alternating gradient descent method was used. In each iteration of this algorithm, first *U* and $\beta_{i}^{c}$ are fixed to compute *V* and $\beta_{j}^{d}$ and then *V* and $\beta_{j}^{d}$ are fixed to compute *U* and $\beta_{i}^{c}$. Besides, to accelerate the convergence, the AdaGrad algorithm was applied and the details of this algorithm are deposited in the **Supplementary File 3 (Data Sheet 3)**. The objective function in formula 18 is denoted by *Y* and the partial gradients of biases and latent vectors are calculated as follow:

(19) $$\begin{array}{c} \frac{\partial Y}{\partial u_{i}}=\sum_{j=1}^{m}\frac{v_{j}^{T}(1+rq_{ij}-q_{ij})(exp\;(u_{i}v_{j}^{T}+\beta_{i}^{c}+\beta_{j}^{d}))}{\left(1+exp\;(u_{i}v_{j}^{T}+\beta_{i}^{c}+\beta_{j}^{d})\right)}-rq_{ij}v_{j}^{T}+(\lambda_{c}u_{i}+\alpha H_{ij}^{c}u_{i})\\ \frac{\partial Y}{\partial v_{j}}=\sum_{i=1}^{n}\frac{u_{i}(1+rq_{ij}-q_{ij})(\text{exp}\;(u_{i}v_{j}^{T}+\beta_{i}^{c}+\beta_{j}^{d}))}{\left(1+exp\;(u_{i}v_{j}^{T}+\beta_{i}^{c}+\beta_{j}^{d})\right)}-rq_{ij}u_{i}+(\lambda_{d}v_{j}+\beta H_{ij}^{d}v_{j})\\ \frac{\partial Y}{\partial \beta_{i}^{c}}=\sum_{j=1}^{m}\frac{\left(1+rq_{ij}-q_{ij})(\text{exp}\;(u_{i}v_{j}^{T}+\beta_{i}^{c}+\beta_{j}^{d})\right)}{\left(1+\text{exp}\;(u_{i}v_{j}^{T}+\beta_{i}^{c}+\beta_{j}^{d})\right)}-rq_{ij}\\ \frac{\partial Y}{\partial \beta_{j}^{d}}=\sum_{i=1}^{n}\frac{\left(1+rq_{ij}-q_{ij})(\text{exp}\;(u_{i}v_{j}^{T}+\beta_{i}^{c}+\beta_{j}^{d})\right)}{\left(1+\text{exp}\;(u_{i}v_{j}^{T}+\beta_{i}^{c}+\beta_{j}^{d})\right)}-rq_{ij} \end{array}.$$

Once the latent matrices *U* and *V* and the biases $\beta_{i}^{c}$ and $\beta_{j}^{d}$ have been learned, the probability of sensitivity cell line *i* to drug *j* can be estimated by logistic function in formula 2. Since in our model, the importance of the positive observations and negative observations are the same, we set *r* = 1 in this logistic function.

#### Prediction

When a new cell line is given, its information of IC50 of the drugs is unknown and *Sim_IC50_* matrix values cannot be calculated, while it must be calculated to predict the latent vectors of this new cell line. In this section, we introduced a classification model for predicting *t*-most nearest neighbors by using the similarity values between cell lines which are obtained from gene expression profile, copy number alteration and single-nucleotide mutation information. The purpose of this model is to find *t*-most nearest neighbors for the new cell line and then to estimate the latent vector for this new cell line based on average of latent vectors of its neighbors. After obtaining the latent vector, we can predict the IC50 values across all drugs for the new cell line. For training the model, 10-fold cross validation technique is used on cell line dataset, so the dataset was partitioned into 10 equal-sized subsets, nine subsets were used as the train set for learning this classification model. A single subset was used as the test set to predict the *t*-most nearest neighbors for each cell line of this set.

In this classification model, the amounts of *Sim_IC50_* matrix of train set were converted to 0 or 1. To do this, the values of each row of the matrix are sorted in descending order and then *t*-largest values are set to 1 and remaining values are set to 0. Among the methods available for classification, we chose “Decision Tree Classifier” method. It is one of the predictive modeling approaches that used tree models to predict the value of a target variable based on several input features. Where leaves represent class labels and branches denote conjunctions of features that lead to those class labels. Learned trees can be represented as sets of if-then rules. Decision tree classifier is a heuristic and nonbacktracking search through the space of all possible decision trees. The main idea of decision tree classification is recursively partition data into subgroups. The functionality of decision tree classification is as follows: Polat and Güneş (2007)

- Choosing an attribute and formulating a logical attribute test.
- Branching on each test result, transferring subset of examples (training information) to the appropriate child node to satisfy that result.
- Running each child’s node recursively.
- The end rule indicates when a leaf node is to be declared.

For decision tree classifier, the three features of train set, *Sim_exp_*, *Sim_cnv_*, and *Sim_mut_*, are considered as input and 0 or 1 value of each pair (*c_i_,c_j_*) are considered as output and then as the classifier train. If the number of predicted nearest neighbors for a cell line was less than *t*, we considered them as nearest neighbors for this cell line. If this number was greater than *t*, *t* neighbors were selected randomly. Finally, *u_i_* was estimated as the average of latent vectors of neighbors of the new cell line *c_i_*.

When the latent vector of the new cell line is predicted, the probabilities that this cell line is sensitive to drugs are estimated. Eventually, a threshold on probabilities to assign sensitive or resistance class to each cell line-drug pair is applied. So if the predicted value is lower than this threshold for a cell line-drug pair, the resistance class is assigned to it; otherwise, it is labeled as a sensitive class.

## Result

We empirically evaluate our proposed approach and compare it against some of the state-of-the-art methods. This section first describe evaluation criteria and then demonstrate the performance of DSPLMF method.

### Evaluation Criteria

To evaluation the performance of DSPLMF method, the 10-fold cross-validation Was performed and this process was repeated 30 times. The mean of following criteria was obtained in the 30 times and it was used as the final criteria to evaluate the predictive performance of the methods.

(20) $$\begin{array}{c} \mathrm{Accuracy}=\frac{\mathrm{TP}+\mathrm{TN}}{\mathrm{TP}+\mathrm{FP}+\mathrm{TN}+\mathrm{FN}}\\ \mathrm{Recall}=\frac{\mathrm{TP}}{\mathrm{TP}+\mathrm{FN}}\\ \mathrm{Precision}=\frac{\mathrm{TP}}{\mathrm{TP}+\mathrm{FP}}\\ \mathrm{Specificity}=\frac{\mathrm{TN}}{\mathrm{TN}+\mathrm{FP}}\\ F_{1}\mathrm{Score}=\frac{2\mathrm{TP}}{2\mathrm{TP}+\mathrm{FP}+\mathrm{FN}}\\ \mathrm{MCC}=\frac{\mathrm{TP}*\mathrm{TN}-\mathrm{FP}*\mathrm{FN}}{\sqrt{\left(\mathrm{TP}+\mathrm{FP})(\mathrm{TP}+\mathrm{FN})(\mathrm{FP}+\mathrm{TN})(\mathrm{FN}+\mathrm{TN}\right)}} \end{array}$$

where *TP* or true positive prediction is the number of cell lines labeled with sensitivity and predicted as sensitivity. *TN* or true negative is the number of cell lines labeled with resistance and predicted as resistance. *FP* or false positive is the number of cell lines labeled with resistance and predicted as sensitivity. *FN* or false negative is the number of cell lines labeled with sensitivity and predicted as resistance.

In addition to the above metrics, we used area under the receiver operating characteristic curve (*AUC*), which is one of the most important evaluation metrics for checking the performance of any classification model. This metric was calculated for the methods.

### Comparison With the State-of-the-Art-Methods

To demonstrate the effectiveness of our method, we compared the predictive performance of the proposed model against the state-of-the-art-methods such as naive Bayes Barretina et al. (2012), SVM-RFE Dong et al. (2015), FSelector Soufan et al. (2015), CaDRReS Suphavilai et al. (2018), AutoBorutaRF Lu et al. (2019), and the AutoHidden method, which is constructed based on the hidden layer of the autoencoder in AutoBorutaRF method as features Lu et al. (2019).

All the methods mentioned above are classification models except the CaDRReS, since this method predicted IC50 values as output, a threshold was applied for its output. So if the value predicted for a cell line-drug pair is smaller than this threshold, the resistance class was assigned to it; otherwise, it was labeled with sensitive class. The median of the IC50 values was chosen as the best threshold for this algorithm. The results of the mentioned methods on two datasets GDSC and CCLE are shown in **Tables 2** and **3**, and the bold number represents the best result. The results of **Table 2** show that the value of *Accuracy* criterion by DSPLMF has increased by 0.03 compared to the result of the best algorithm, AutoBorutaRF. Furthermore, the value of *Recall*, *F_1_Score*, *MCC*, and *AUC* criteria have increased by 0.10, 0.05, 0.06, and 0.05 compared to the best algorithm. Only in the case of the *Specificity* criterion, the naive Bayes method performs significantly better than the other methods. The reason is that this method has predicted zero class data for most of the data, and by looking at the result of other criteria, such as *Accuracy*, *Recall*, and *F_1_Score* for this method, we can see that this method does not predict sensitive class data very well. The results of **Table 3** are the same as those in the previous table, except that the best result for the *AUC* criterion belongs to the AutoBorutaRF method, demonstrating the effectiveness of this method. The best result for the *Specificity* criterion belongs to the AutoHidden method; the low performance of other criteria indicates that this method is weak in predicting sensitive data. In general, the results of these two tables show that the DSPLMF significantly outperforms other methods. Thus, it is evident our method able to find much more useful features for drug response prediction rather than other methods. Overall, DSPLMF improvement on the GDSC dataset is stronger.

**Table 2:** Prediction performance of the different algorithms based on seven criteria on Genomics of Drug Sensitivity in Cancer (GDSC) dataset.

Method	*Accuracy*	*Recall*	*Precision*	*Specificity*	*F*_1_ *Score*	*MCC*	*AUC*
DSPLMF	**0.682**	**0.750**	**0.671**	0.615	**0.702**	**0.373**	**0.760**
CaDRReS	0.541	0.540	0.547	0.546	0.549	0.110	0.510
AutoBorutaRF	0.653	0.652	0.646	0.654	0.650	0.310	0.711
naive Bayes	0.610	0.424	0.590	**0.796**	0.494	0.247	0.679
SVM-RFE	0.594	0.579	0.589	0.609	0.585	0.191	0.515
FSelector	0.606	0.617	0.593	0.595	0.606	0.215	0.647
AutoHidden	0.578	0.557	0.571	0.598	0.565	0.158	0.609

The 10-fold cross validation is applied on the evaluation metrics and the mean value of them is used as criteria for comparison.

**Table 3:** Prediction performance of the different algorithms based on seven criteria on Cancer Cell Line Encyclopedia (CCLE) dataset.

Method	*Accuracy*	*Recall*	*Precision*	*Specificity*	*F*_1_ *Score*	*MCC*	*AUC*
DSPLMF	**0.770**	**0.723**	**0.636**	0.772	**0.677**	**0.481**	0.776
CaDRReS	0.671	0.353	0.493	0.830	0.412	0.202	0.501
AutoBorutaRF	0.763	0.656	0.594	0.813	0.624	0.452	**0.821**
naive Bayes	0.683	0.332	0.406	0.919	0.366	0.275	0.779
SVM-RFE	0.728	0.428	0.631	0.812	0.523	0.296	0.551
FSelector	0.743	0.506	0.630	0.805	0.563	0.353	0.737
AutoHidden	0.697	0.133	0.201	**0.950**	0.356	0.219	0.706

The 10-fold cross validation is applied on the evaluation metrics and the mean value of them is used as criteria for comparison.

### Performance of the Novel Regularization Approach

To evaluate the improvement of the logistic matrix factorization method by applying the novel regularization approach, we compared the predictive performance of the DSPLMF model against the logistic matrix factorization method without the novel regularization approach. In this model, the classification method for predicting t-most nearest neighbors for each new cell line by using the similarity values between cell lines which are obtained from gene expression profile, copy number alteration and single-nucleotide mutation information, is not applied. The result of the above algorithm based on seven criteria on GDSC and CCLE datasets is calculated, and the 10-fold cross-validation is applied on the evaluation metrics, and the mean value of them is used as criteria for comparison. The results of **Tables 2** and **4** show that the value of *Accuracy* criterion by DSPLMF on GDSC dataset has increased by 0.10 compared to the result of the logistic matrix factorization method without the novel regularization approach. Furthermore, the value of *Recall*, *Precision*, *Specificity*, *F_1_Score*, *MCC*, and *AUC* criteria have increased by 0.04, 0.10, 0.17, 0.07, 0.21, and 0.14 compared to this algorithm. The results of **Tables 3** and **4** show that the value of *Accuracy* criterion by DSPLMF on CCLE dataset has increased by 0.10 compared to the result of the logistic matrix factorization method without the novel regularization approach. Furthermore, the value of *Recall*, *Precision*, *Specificity*, *F_1_Score*, *MCC*, and *AUC* criteria has increased by 0.05, 0.11, 0.10, 0.09, 0.16, and 0.10 compared to this algorithm. So, using of the classification method for predicting t-most nearest neighbors of each new cell line in logistic matrix factorization algorithm, will increase the performance by 10%.

**Table 4:** Prediction performance of the logistic matrix factorization method without the novel regularization approach based on seven criteria on Cancer Cell Line Encyclopedia (CCLE) and Genomics of Drug Sensitivity in Cancer (GDSC) datasets.

Dataset	*Accuracy*	*Recall*	*Precision*	*Specificity*	*F*_1_ *Score*	*MCC*	*AUC*
GDSC	0.580	0.713	0.571	0.442	0.630	0.168	0.626
CCLE	0.672	0.673	0.523	0.670	0.582	0.328	0.671

The 10-fold cross validation is applied on the evaluation metrics and the mean value of them is used as criteria for comparison.

### Specific Tissue of Cell Line Type

**Figure 3:**
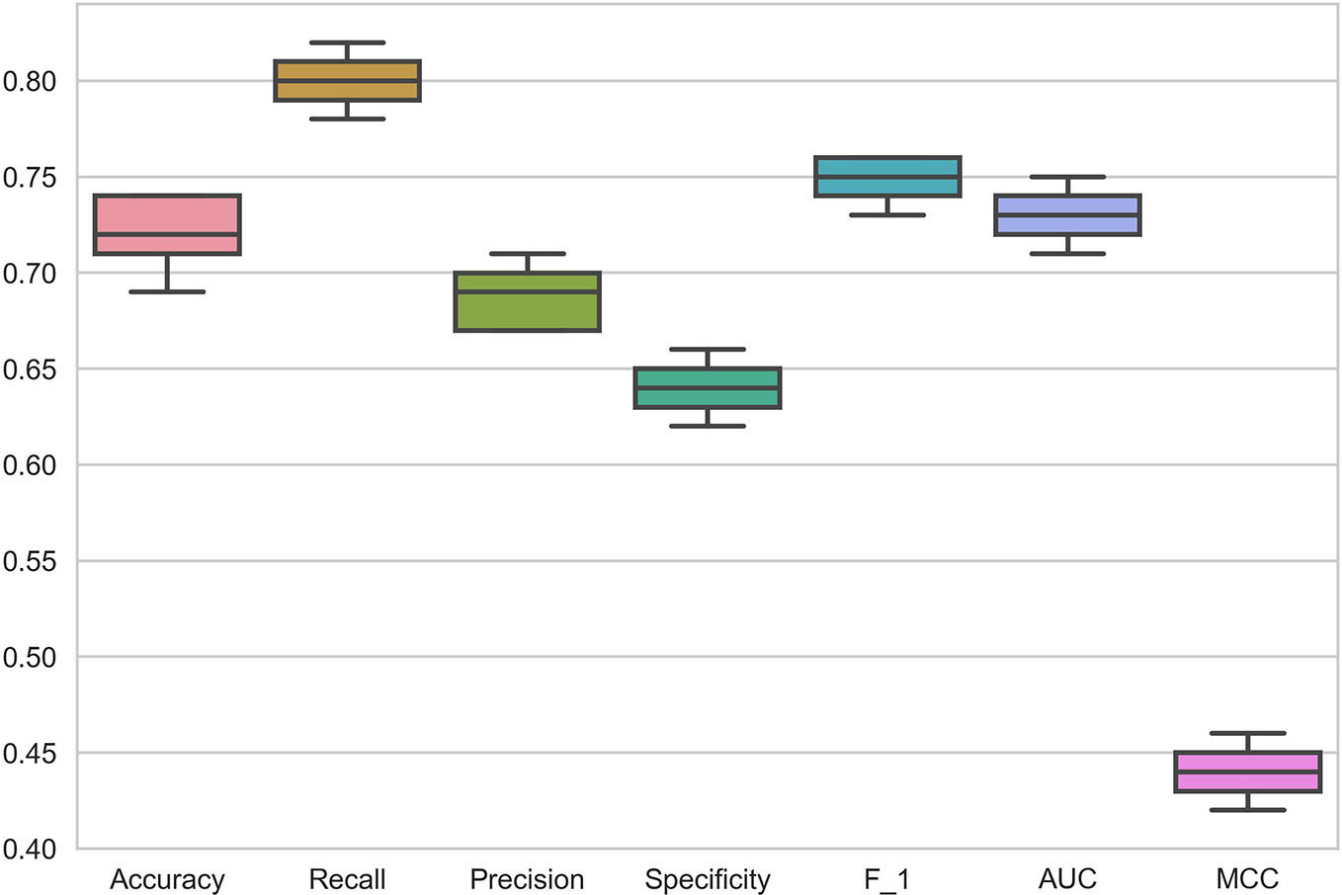
Box Plots of seven criteria on haematopoietic cell lines in Genomics of Drug Sensitivity in Cancer (GDSC) dataset to show the prediction performance of DSPLMF method.

The data in the GDSC dataset is related to different cancers. To demonstrate the performance of DSPLMF method on cancer tissue type, 73 hematopoietic cell lines and 98 drugs from GDSC dataset are considered. This specific type of cell lines are used to train the proposed model and predicted responses for the drugs based on this tissue type. **Figure 3** shows the results of all mentioned criteria on these cell lines for the DSPLMF method using 30 times 10-fold cross-validation. The mean of these values are shown in **Table 5**. As the table shows, if the algorithm is specifically run on a particular type of cancer, it would be expected to yield better results than when considering different types of cancer. These results indicate that DSPLMF can also achieve consistent performance on a specific type of cancer.

**Table 5:** Prediction performance of DSPLMF method on haematopoietic cell lines based on seven criteria on Genomics of Drug Sensitivity in Cancer (GDSC) dataset.

Method	*Accuracy*	*Recall*	*Precision*	*Specificity*	*F*_1_ *Score*	*MCC*	*AUC*
DSPLMF	0.721	0.800	0.690	0.645	0.750	0.441	0.730

The 10-fold cross validation is applied on the evaluation metrics and the mean value of them is used as criteria for comparison.

### Correlation Between Predicted and Observed Responses Values

**Figure 4:**
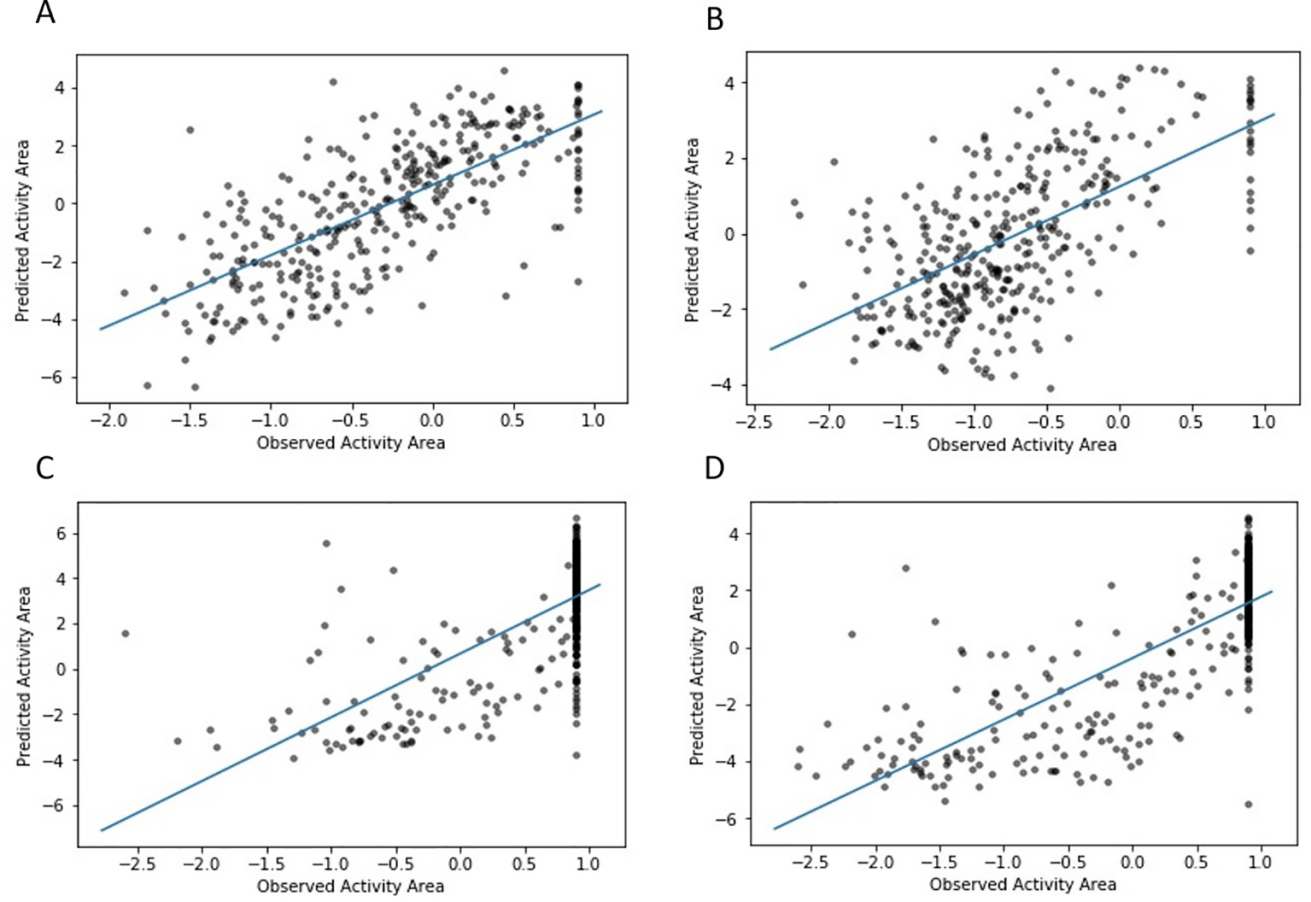
Correlations between observed and predicted activity areas using DSPLMF method for CCLE cell lines across four drugs. **(A)** shows the scatter plot of observed and predicted drug responses for Topotecan with 0.71 as Pearson Correlation. **(B)** shows the scatter plot of observed and predicted drug responses for 17-AAG with 0.60 as Pearson Correlation. **(C)** shows the scatter plot of observed and predicted drug responses for AZD6244 with 0.68 as Pearson Correlation. **(D)** shows the scatter plot of observed and predicted drug responses for PD-032590 with 0.79 as Pearson Correlation.

For further evaluation and to demonstrate the performance of the proposed algorithm, the scatter plots of observed versus predicted responses values for four drugs in CCLE are illustrated in **Figure 4**. The values predicted by our model are probabilities that cell lines are sensitive to the drugs. For calculation correlation between predicted and observed responses values, the values $\left(u_{i}v_{j}^{T}+\beta_{i}^{c}+\beta_{j}^{d}\right)$ in Formula 2 as the predicted IC50 values for cell line *c_i_* and drug *d_j_* were used. As the plots indicate, there is a high correlation between observed and predicted response values. The scatter plots of all 24 drugs in the CCLE dataset are illustrated in the **Supplementary File 2 (Data Sheet 2: Figures S1–S4)**.

### Learning Hyperparameters

For tuning hyperparameters, GDSC dataset has been used, and the obtained hyperparameters are considered for both datasets. The 10-fold cross-validation procedure is applied on GDSC and hyperparameters are chosen empirically by maximizing the summing up of the Accuracy, Recall, Precision, Specificity, *F_1_*Score, and MCC criteria. For each set of hyperparameters, the whole 10-fold process is repeated 30 times and the average value of the above summing has been calculated. Since the search space of hyperparameters values is large, a grid-search procedure for choosing the hyperparameters was applied.

The dimension of latent space, *L*, was selected between 1 and 98, the number of drugs. The number of KNNs for building *N_k_*(*c_i_*) in equation 13 and the number of *t*-nearest neighbors in prediction section, were selected from 1 to 50 by step 2. The impact factors of nearest neighbors α and β in equations 15 and 16 were picked from {2^–5^, 2^–4^,…, 2^2^} and the variance controlling parameters, λ*_c_* and λ*_d_*, were chosen from{2^–5^, 2^–4^,…, 2^1^}. The γ, λ, *ϕ* and *ψ* parameters represent the importance of each similarity measure between cell lines in formula 1 and were selected from 1 to 10. Threshold parameter applied on equation 2 for determining the label of the class for each new cell line *c_i_*, and was picked from 0.1 to 1 by step 0.1, and the best accuracy of the result is obtained by threshold=0.6.

In **Table 6**, the learned hyperparameters using GDSC dataset is shown. For both datasets, these tuned hyperparameters are used to design the model, except to *L*, that is calculated for CCLE dataset separately and for this dataset it is set as 23.

**Table 6:** Learned hyperparameters of DSPLMF method based on Genomics of Drug Sensitivity in Cancer (GDSC) dataset.

Hyperparameters	*L*	*k*	*t*	*λ*_c_	*λ*_d_	*α*	*β*	*λ*	*γ*	*ϕ*	*ψ*	*Threshold*
value	95	20	20	0.6	0.6	0.5	0.1	1	1	1	3	0.6

## Discussion

### Cell Line Subtypes in Latent Space

**Figure 5:**
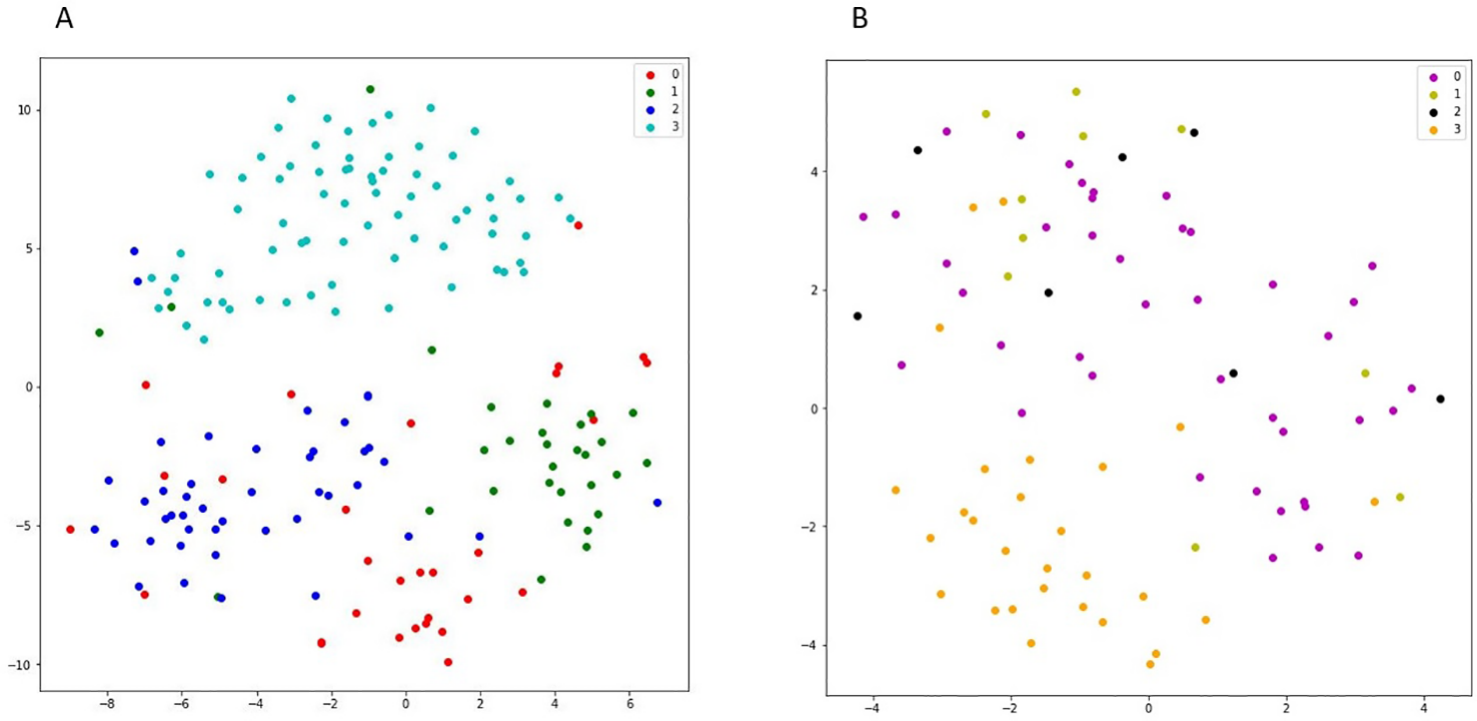
**(A)** shows the t-SNE plot of latent space corresponding to four different cancer subtypes in GDSC dataset. In this figure, red points show the latent vectors of breast cancer and green, dark blue, and light blue points show the latent vectors of COREAD, central nervous system, and haematopoietic and lymphiod tissue, respectively. **(B)** shows the t-SNE plot of latent space corresponding to different lung cancer subtypes in Genomics of Drug Sensitivity in Cancer (GDSC) dataset. In this figure, purple points show the latent vectors of adenocarcinoma and light green, black and orange points show the latent vectors of large cell, squamous cell, and small cell carcinoma, respectively.

We used 555 cell lines from different cancerous tissue types in GDSC dataset. For representing the higher similarity between latent vectors ${\tilde{u}}_{i}$ of the cell lines from the same tissue type rather than the cell lines from different tissue types, the t-SNE plot for some tissue types of cancer cell lines is shown in **Figure 5**. Top five most frequent tissue types including, breast, central nervous system, hematopoietic and lymphoid tissue, COREAD, and lung cancer were considered. As it can be seen from **Figure 5** (A), the embedded latent vectors of the cell lines with the same tissue type are located closer than the cell lines with diverse tissue types. This suggests that the proposed method assigned more similar latent vector to cell lines with the same tissue type. In the following, we consider an example of some latent vectors and the similarities between them: Let *v_1_*, *v_2_* and *v_3_* are three latent vectors obtained DSPLMF method of length 95 corresponding to Breast cancer cell line *BT* − 20, Breast cancer cell line *BT* − 549 and hematopoietic cancer cell line *CA*46, respectively. *v_1_* = [0.01, 0.23, −0.14,…, 0.12]_1x95_, *v_2_* = [0.17,0.67, −0.1,…,0.34]_1x95_ and *v_3_* = [0.89, −0.9, 0.55,…, −0.17]_1x95_. Similarity (*v_1_,v_2_*) = 0.78, Similarity (*v_1_,v_2_*) = 0.13 and Similarity(*v_2_,v_3_*) = 0.04. As the results show, two vectors belonging to the same tissue types are more similar than two vectors that belong to two different tissue types. Also, in the t-SNE plot, these two vectors belonging to the same tissue types are closer than two vectors that belong to two different tissue types.

In **Figure 5B**, the latent vectors of different subtypes of lung cancer were considered. These different subtypes are: adenocarcinoma, large cell, squamous cell, and small cell carcinoma. In this figure, the closeness of vectors corresponding to cell line of the same subtype in this cancer justifies the efficiency of obtained latent vectors.

### Investigation Drug-Pathway Association

For inferring drug-pathway associations, the heatmap of Pearson correlation between predicted drug responses and pathway activity scores similar to Suphavilai et al. (2018) is used. We considered 50 Biocarta pathway gene sets from MSigDB Liberzon et al. (2011), and pathway activity scores for CCLE cell lines were calculated as follows:

Let *PW* is a pathway and G(PW) = {*g*_1_,*g*_2_,…, *g_r_*} is the set of genes corresponding to pathway *PW*. Let fold-change value of *g_i_* in cell line *c_j_* is *x_ij_*, which is obtained by:

(21) $$\begin{array}{c} x_{ij}=Log_{2}(expression\;intensity\;of\;g_{i}\;in\;cell\;line\;c_{j})-median(Log_{2}(expression\;intensity\;of\;g_{i}\;in\;all\;cell\;lines)) \end{array}$$

Pathway activity score of pathway *PW* for cell line *c_j_*, *PAS_j_(PW)* was calculated by formula 22.

(22) $$PAS_{j}(PW)=\sum_{i=1}^{r}x_{ij}$$

Pathway activity score of *PW* for all cell line, *PAS*(*PW*), are considered as the vector *PAS(PW)* = [*PAS_1_(PW), PAS_2_(PW),…, PAS_n_(PW)*], where *n* is the numbers of cell lines. Also, the predicted drug responses by DSPLMF for each drug were considered as the vector *IC*50*_predicted_* = [*IC_1_, IC_2_,…,IC_n_*].

**Figure 6:**
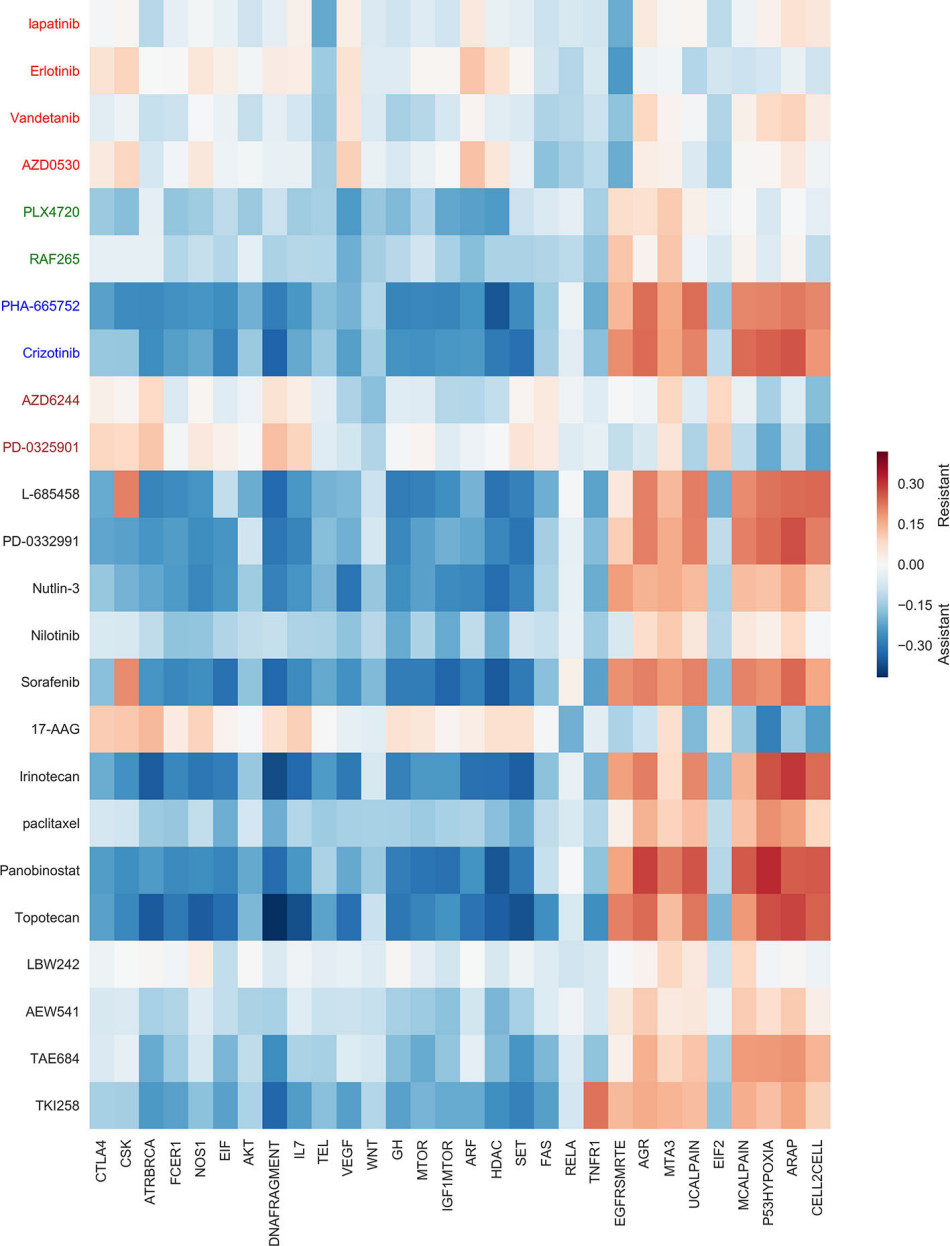
Drug-pathway association based on Cancer Cell Line Encyclopedia (CCLE) dataset. For visualization, 30 Biocarta pathways across 24 drugs were selected. Negative and positive correlations between pathway activity and drug sensitivity scores are denoted as being “assistant” and “resistant” associations, respectively. The blue color is represented the assistance and the red color is represented the resistance.

Then, the association between drug *d_j_* and pathway *PW* is computed by the Pearson correlation between *IC50_predicted_* for drug *d_j_* and *PAS*(*PW*). A positive correlation indicates that a pathway plays a role in drug resistance and negative correlation demonstrated that a pathway is important in drug sensitivity. The result of the Pearson correlation of 30 pathway gene sets and 24 drugs of CCLE dataset is shown in **Figure 6** and the result of 20 other pathways is represented in the **Supplementary File 1 (Data Sheet 1)**. In this figure, the blue is represented the assistance and the red is represented the resistance case. Below, we investigated several instances that indicates consistency between the result of calculated Pearson correlation and previous studies and researches.

- The activation score of the HDAC (Histone deacetylases) pathway is negatively correlated (assistant association) with predicted IC50 value of some drugs such as Panobinostat. These observations were consistent with two studies, showing that the Panobinostat can inactive HDAC pathway De Marinis et al. (2013); Yee and Raje (2018).
- We observed the RELA (Acetylation and Deacetylation of RelA in The Nucleus) pathway had an assistant association with the 17-AAG (HSP90 inhibitor) drug. The RELA gene is one member of the NF-kB family and two important roles of the RELA are the transcriptional regulation and NF-kB signed transduction. Since the 17-AAG drug affects the NF-kB activity, it also affects the RELA gene and RELA pathway Thangjam et al. (2014).
- The activation score of the *EGFR* − *SMRTE* pathway was negatively correlated with predicted IC50 value of four EGFR inhibitors drugs, namely, Lapatinib, Erlotinib, Vandetanib, and AZD0530. These observations matched the previous study that denoted the amplification of the EGFR gene is correlated with a high response to EGFR inhibitors Normanno et al. (2006). Moreover, the predicted IC50 values of the Crizotinib (ALK-inhibitor) were positively correlated with the activity score of this pathway and this issue was confirmed in the previous studies Sasaki et al. (2011).
- The MTA3 (Downregulated of MTA-3 in ER-negative Breast Tumors) pathway was associated (positively correlated) with two predicted IC50 vectors belong to L-685458(gamma-secretase) and PD-0332991(CDK4/6) drugs. Therefore, the cell lines with inactivated MTA3 pathway tend to sensitive to these two drugs Suphavilai et al. (2018).
- The VEGF-Hypoxia-Angiogenesis (VEGF) pathway was assistance associated with two RAF inhibitors drugs, namely, PLX4720 and RAF265 drugs that were verified in the previous researches. One of these studies considered inducing the VEGF expression by Raf promotes angiogenesis and blocking *RAF*/*MEK*/*ERK* pathway by RAF inhibitors McCubrey et al. (2007). Moreover, the activity score of the VEGF pathway was negatively correlated with Sorafenib drug Liu et al. (2006).
- The activity score of the mTOR Signaling Pathway that is a central regulator of metabolism and physiology was negatively correlated with predicted Ic50 vector of some drugs such as Panobinostat. Various preclinical studies have been performed to combine panobinostat with several drugs as mTOR inhibitor Singh et al. (2016).
- It has been shown that c-met inhibitor drugs such as PHA-665752 and Crizotinib can inhibit WNT pathway activity in tumour cells. We observed the activity score of this pathway was negatively correlated with predicted IC50 vectors of these drugs Tuynman et al. (2008); Zhang et al. (2018).
- The assistant association was observed between *L* − 685458 drug and IGF-1 MTOR pathways. These observations were also reported by Shih et al Shih and Wang (2007).
- We observed that the MEK inhibitors such as *AZD*6244 and *PD* − 0325901 were positively correlated with activity scores for the EIF2 pathway. Therefore, as mentioned in the previous researches, the cell lines with inactivated EIF2 p athway were sensitive to these drugs Quevedo et al. (2000); Liberzon et al. (2011).

### Conclusion

In this work, we introduce a novel method for cancer drug sensitivity prediction based on a recommender system approach. A logistic matrix factorization is applied to predict the extent to which a cell line is sensitive to a drug. The advantage of this method is to obtain latent features of cell lines and drugs for better prediction performance. Since the similarity information of cell lines and drugs can improve higher predictive power, some information such as gene expression profile, copy number alteration and single-nucleotide mutation data for cell lines and Chemical structures of drugs are used.

To demonstrate the validity of DSPLMF method for identifying drug response 10-fold cross validation on CCLE and GDSC datasets are performed. The comparison of DSPLMF with six other the state-of-the-art prediction methods showed that DSPLMF outperformed other methods. The results indicated that the proposed method was able to uncover much more effective features than the other methods for drug response prediction.

## Data Availability Statement

The source code of proposed method and *Datasets* folder for GDSC and CCLE datasets as input data are available in https://github.com/emdadi/DSPLMF and **Supplementary File 4 (Data Sheet 4)**.

## Author Contributions

AE designed the algorithm, performed the experiments, and wrote the main manuscript text and the programming codes. CE conducted the experiments and analyzed the results. All authors reviewed the manuscript.

## Conflict of Interest

The authors declare that the research was conducted in the absence of any commercial or financial relationships that could be construed as a potential conflict of interest.

## Supplementary Material

Supplementary File 1 (Data Sheet 1) Results of drug pathway association on CCLE dataset.

Supplementary File 2 (Figures S1–S4) The scatter plots of all 24 drugs in the CCLE dataset.

Supplementary File 3 (Data Sheet 3) AdaGrad Algorithm.

Supplementary File 4 (Data Sheet 4) Implementation Codes.
