# Supplementary material for: DSPLMF: A Method for Cancer Drug Sensitivity Prediction Using a Novel Regularization Approach in Logistic Matrix Factorization
Source: Front Genet. 2020 Feb 27;11:75. doi: 10.3389/fgene.2020.00075 (PMC7056895; doi:10.3389/fgene.2020.00075)
Supplement: Supplementary File 2 (Figures S1–S4) — The scatter plots of all 24 drugs in the CCLE dataset. [file DataSheet_2.pdf]

Supplementary Material

1 SUPPLEMENTARY DATA

Supplementary File 1 . Results of drug pathway association on CCLE dataset. Supplementary File 2 . AdaGrad Algorithm. Supplementary File 3 . Implementation Codes.

2 SUPPLEMENTARY TABLES AND FIGURES

2.1 Figures

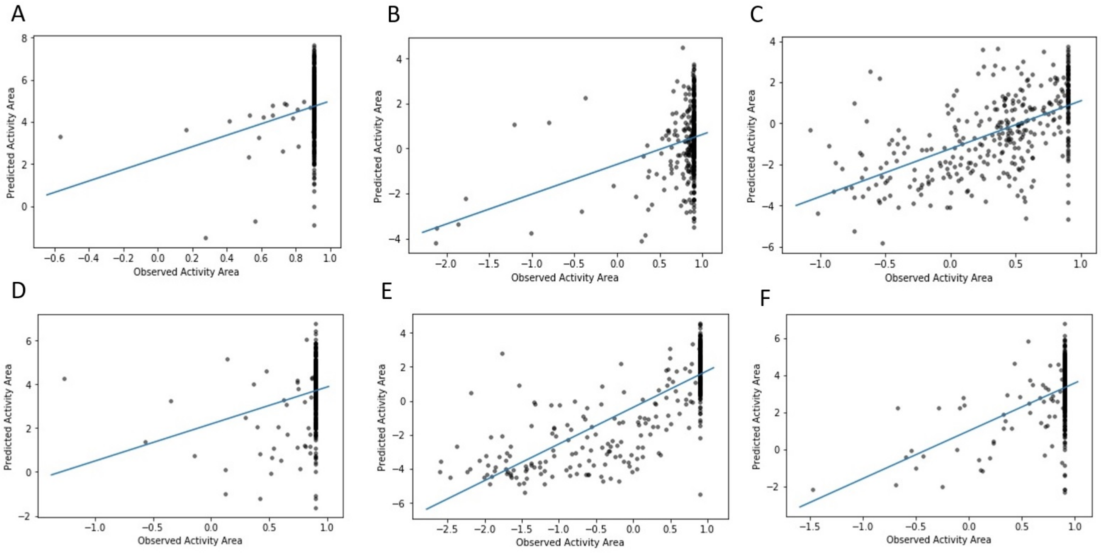

Figure S1. Correlations between observed and predicted activity areas using introduced method, **(A)** Nutlin-3 (Pearson Correlation:0.21), **(B)** Nilotinib (Pearson Correlation:0.32), **(C)** RAF265 (Pearson Correlation:0.58), **(D)** PHA-665752 (Pearson Correlation:0.23), **(E)** PD-0325901 (Pearson Correlation:0.79), **(F)** PLX4720 (Pearson Correlation:0.45)

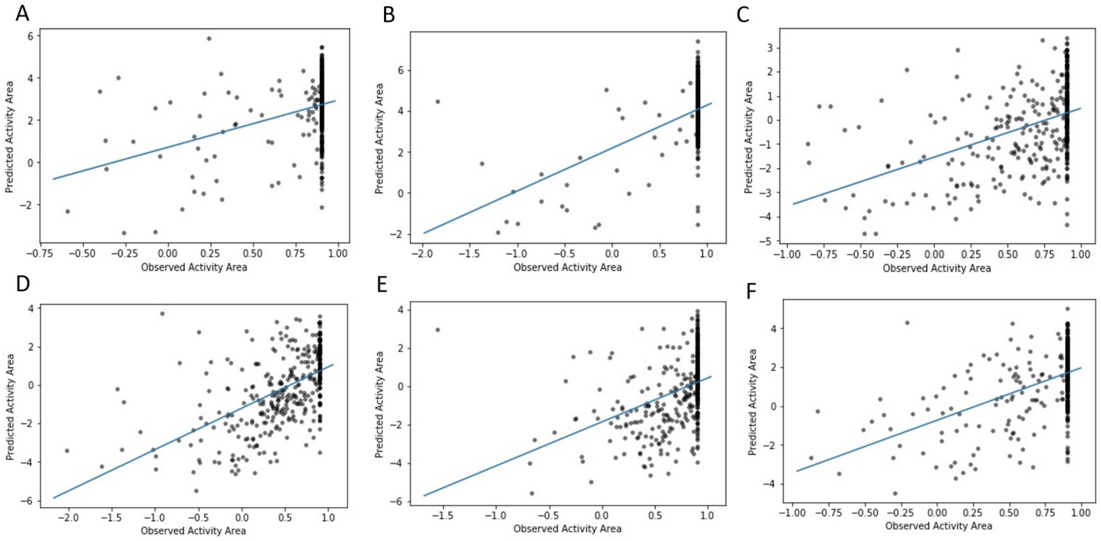

Figure S2. Correlations between observed and predicted activity areas using introduced method, **(A)** PD-0332991 (Pearson Correlation:0.36), **(B)** LBW242 (Pearson Correlation:0.52), **(C)** AEW541 (Pearson Correlation:0.46), **(D)** TAE684 (Pearson Correlation:0.55), **(E)** TKI2581 (Pearson Correlation:0.40), **(F)** AZD0530 (Pearson Correlation:0.51)

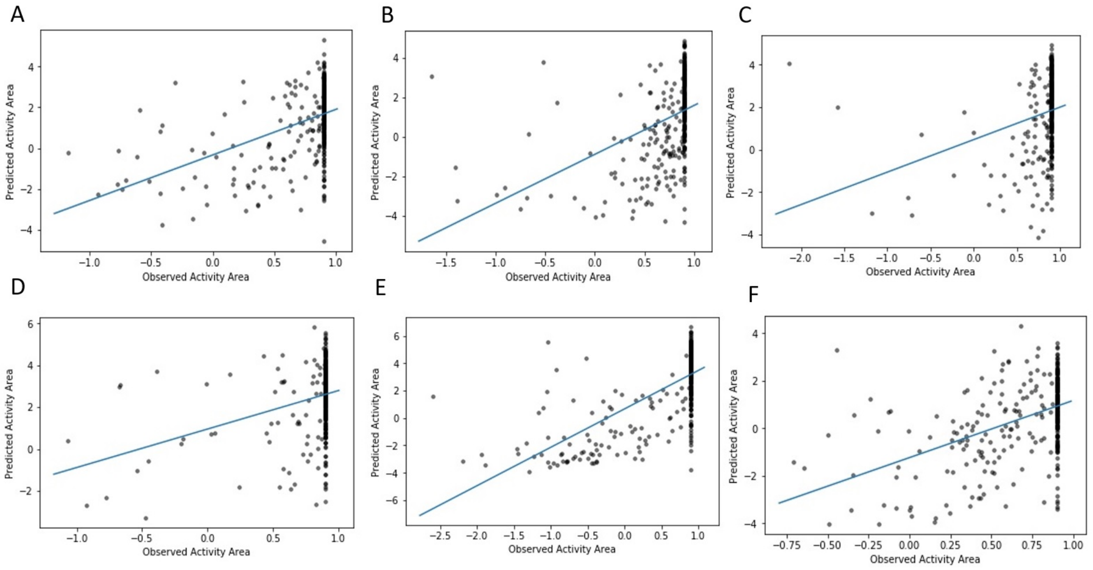

Figure S3. Correlations between observed and predicted activity areas using introduced method, **(A)** lapatinib (Pearson Correlation:0.46), **(B)** Crizotinib (Pearson Correlation:0.43), **(C)** Sorafenib (Pearson Correlation:0.28), **(D)** L-685458 (Pearson Correlation:0.32), **(E)** AZD6244 (Pearson Correlation:0.68), **(F)** Vandetanib (Pearson Correlation:0.47)

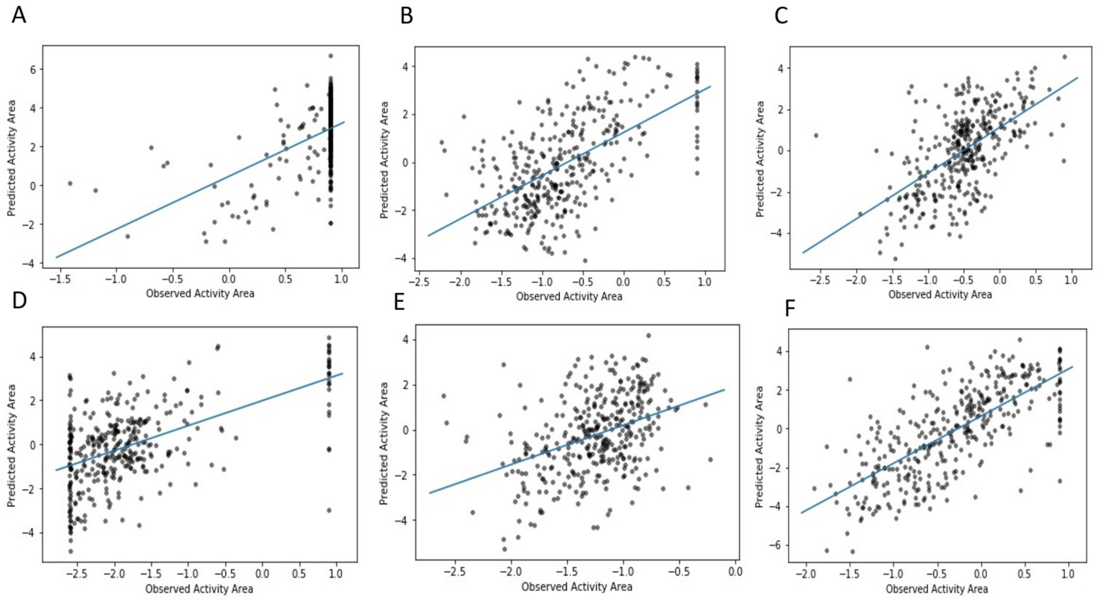

Figure S4. Correlations between observed and predicted activity areas using introduced method, **(A)** Erlotinib (Pearson Correlation:0.51), **(B)** 17-AAG (Pearson Correlation:0.60), **(C)** Irinotecan (Pearson Correlation:0.59), **(D)** paclitaxel (Pearson Correlation:0.56), **(E)** Panobinostat1 (Pearson Correlation:0.40), **(F)** Topotecan (Pearson Correlation:0.71)
