# Supplementary material for: DSPLMF: A Method for Cancer Drug Sensitivity Prediction Using a Novel Regularization Approach in Logistic Matrix Factorization
Source: Front Genet. 2020 Feb 27;11:75. doi: 10.3389/fgene.2020.00075 (PMC7056895; doi:10.3389/fgene.2020.00075)
Supplement: Supplementary File 3 (Data Sheet 3) — AdaGrad Algorithm. [file DataSheet_3.pdf]

AdaGrad Algorithm

Adagrad is an algorithm for gradient-based optimization that adapts the learning rate of the gradient step size. The learning rate reflects how much we allow the parameter to follow the opposite direction of the gradient estimate. Since this rate could be very difficult to set because if we set it too large, the parameter will move throughout the function and may never result in any acceptable loss. Otherwise, if we set it too small, it will be very slow to update the parameter and it will take a long time to achieve an appropriate loss.

Previously, we performed an update for all parameters θ and every parameter θ_i used the same learning rate η. we use g_t to denote the gradient at time step t $g_{t,i}$ is then the partial derivative of the objective function to the parameter θ_i at time step t:

$$g_{t,i} = \nabla_\theta J(\theta_{t,i}) \tag{S1}$$

Adagrad uses a different learning rate and modifies it for every parameter θ_i at every time step t based on the past gradients that have been computed for θ_i:

$$\theta_{t+1,i} = \theta_{t,i} - \frac{\eta}{\sqrt{G_{t,ii} + \epsilon}} \cdot g_{t,i} \tag{S2}$$

$G_t \in \mathbb{R}^{d \times d}$ is a diagonal matrix where each diagonal element (i, i) is the sum of the squares of the gradients θ_i up to time step t, while ϵ is a smoothing term that avoids division by zero. One of main benefits of Adagrad is that it eliminates the need to manually tune the learning rate.
