# Supplementary material for: DSPLMF: A Method for Cancer Drug Sensitivity Prediction Using a Novel Regularization Approach in Logistic Matrix Factorization
Source: Front Genet. 2020 Feb 27;11:75. doi: 10.3389/fgene.2020.00075 (PMC7056895; doi:10.3389/fgene.2020.00075)
Supplement: Supplementary File 4 (Data Sheet 4) — Implementation Codes. [file DataSheet_4.pdf]

Main()

-*- coding: utf-8 -*-

"""

Created on 2018

@author: asus

"""

```
from __future__ import division


import os
from sklearn.model_selection import  RepeatedKFold
from function import load_matrices, apply_threshold
from lmf import PSLMF
from function import apply_threshold_median,score_to_exact_rank


from function import compute_evaluation_criteria_across_wholeMatrix
import numpy as np




data_folder = os.path.join(os.path.pardir, 'Datasets') # All dataset's matrices folder



observation_mat, cell_sim,cell_sim_2,observation_mat_IC50,drugMat =load_matrices(data_folder)


seed = [80162,45929]


num_repeats=1 # Number of repetition of 10-fold cross validation



# for GDSC datase:
```

```
#model = PSLMF(c=1, K1=20, K2=20, r=95, lambda_p=0.6, lambda_l=0.6, alpha=0.5,beta=0.1, theta=1.3, max_iter=1000)


#for CCLE dataset:

model = PSLMF(c=1, K1=10, K2=10, r=23, lambda_p=0.6, lambda_l=0.6, alpha=0.5,beta=0.1, theta=1.3, max_iter=1000)


kf = RepeatedKFold(n_splits=10, n_repeats=num_repeats)
ACC_new, F1_score_new= 0.0, 0.0
REC_new=0.0
Specificity=0.0
MCC_new=0.0


AUC=0.0
precision_new=0.0
result=0.0



for train_index, test_index, in kf.split(cell_sim, observation_mat):



  test_location_mat = np.array(observation_mat)

  test_location_mat[train_index] = 0
  train_location_mat = np.array(observation_mat - test_location_mat)

  true_result = np.array(test_location_mat[test_index])


  x = np.repeat(test_index, len(observation_mat[0]))
  y = np.arange(len(observation_mat[0]))

  y = np.tile(y, len(test_index))


  model.fix_model(train_location_mat, train_location_mat,drugMat, cell_sim,cell_sim_2, seed)
```

```
    scores = np.reshape(model.predict_scores(zip(x, y)), true_result.shape)
    pred_Ic50_values=np.reshape(model.predict_scores_2(zip(x, y)), true_result.shape)



    prediction = apply_threshold(scores, 0.6)


    precision_new_o,fold_ACC_new
,fold_F1_score_new,REC_new_output,Specificity_new,MCC_new_output,AUC_new_new_o,fold_loc_
f1 = compute_evaluation_criteria_across_wholeMatrix(true_result, prediction,scores)

    ACC_new+=fold_ACC_new
    F1_score_new+=fold_F1_score_new
    REC_new+=REC_new_output
    Specificity+=Specificity_new
    MCC_new=MCC_new_output+MCC_new
    #AUC_new=AUC_new_o+AUC_new

    precision_new=precision_new_o+precision_new
    AUC=AUC_new_new_o+AUC

    observation_mat_IC50_mat = np.array(observation_mat_IC50)


    RR=np.array(observation_mat_IC50_mat[test_index])

    pred=scores





ACC_new=round(ACC_new/(10*num_repeats),2)
F1_score_new=round(F1_score_new/(10*num_repeats),2)
REC_new=round(REC_new/(10*num_repeats),2)
MCC_new=round(MCC_new/(10*num_repeats),2)
Specificity=round(Specificity/(10*num_repeats),2)


precision_new=round(precision_new/(10*num_repeats),2)
AUC=round(AUC/(10*num_repeats),2)
```

```
            print('ACC_new', ACC_new)
            print('F1_score_new', F1_score_new)
            print('REC_new', REC_new)
            print('Specificity', Specificity)
            print('MCC_new', MCC_new)
            print('AUC', AUC)
            print('precision_new', precision_new)
```

lmf()

```
# -*- coding: utf-8 -*-
"""
Created on  2018
@author: asus
"""
import math
import numpy as np

class PSLMF:

    def __init__(self, c=1, K1=5, K2=20, r=95, theta=1.3, lambda_p=0.6, lambda_l=0.6, alpha=0.4,beta=0.4, max_iter=50):
        self.c = int(c)  # importance level for positive observations
        self.K1 = int(K1) # number of nearest neighbors used for latent matrix construction
        self.K2 = int(K2) # number of nearest neighbors used for score prediction
        self.r = int(r)  # latent matrices's dimension
        self.theta = float(theta) # Gradien descent learning rate
        self.lambda_p = float(lambda_p) # reciprocal of cell lines's variance
        self.lambda_l = float(lambda_l) # reciprocal of drug's variance
        self.alpha = float(alpha) # impact factor of nearest neighbor in constructing method
        self.beta = float(beta)
```

```
    self.max_iter = int(max_iter)

def AGD_optimization(self, seed=None):
    if seed is None:
        self.U = np.sqrt(1/float(self.r))*np.random.normal(size=(self.cell_count, self.r))
        self.V = np.sqrt(1/float(self.r))*np.random.normal(size=(self.locs_count, self.r))
        self.cell_biases = np.sqrt(1/float(self.r))*np.random.normal(size=(self.cell_count, 1))
        self.locs_biases = np.sqrt(1/float(self.r))*np.random.normal(size=(self.locs_count, 1))
    else:
        prng = np.random.RandomState(seed[0])
        prng2= np.random.RandomState(seed[1])
        self.U = prng.normal(loc=0.0, scale=np.sqrt(1/self.lambda_p), size=(self.cell_count, self.r))
        self.V = prng.normal(loc=0.0, scale=np.sqrt(1/self.lambda_l), size=(self.locs_count, self.r))
        self.cell_biases = prng2.normal(size=(self.cell_count, 1))
        self.locs_biases = prng2.normal(size=(self.locs_count, 1))
    cell_sum = np.zeros((self.cell_count, self.U.shape[1]))
    locs_sum = np.zeros((self.locs_count, self.V.shape[1]))
    cell_bias_deriv_sum = np.zeros((self.cell_count, 1))
    locs_bias_deriv_sum = np.zeros((self.locs_count, 1))
    last_log = self.log_likelihood()
    for iter in range(self.max_iter):
        cell,user_bias_deriv = self.deriv(True)
        cell_sum += np.square(cell)
        cell_bias_deriv_sum += np.square(user_bias_deriv)
        vec_step_size = self.theta / (1*np.sqrt(cell_sum))
        bias_step_size = self.theta /(1* np.sqrt(cell_bias_deriv_sum))
        self.cell_biases += bias_step_size * user_bias_deriv
        self.U += vec_step_size * cell
        locs,item_bias_deriv = self.deriv(False)
        locs_sum += np.square(locs)
        locs_bias_deriv_sum += np.square(item_bias_deriv)
```

```
        vec_step_size = self.theta / np.sqrt(locs_sum)
        bias_step_size = self.theta /(1* np.sqrt(locs_bias_deriv_sum))
        self.V += vec_step_size * locs
        self.locs_biases += bias_step_size * item_bias_deriv
        curr_log = self.log_likelihood()
        delta_log = (curr_log-last_log)/abs(last_log)
        if abs(delta_log) < 1e-5:
            break
        last_log = curr_log

def deriv(self, cell):
    if cell:
        vec_deriv = np.dot(self.intMat, self.V)
        bias_deriv = np.expand_dims(np.sum(self.intMat, axis=1), 1)
    else:
        vec_deriv = np.dot(self.intMat.T, self.U)
        bias_deriv = np.expand_dims(np.sum(self.intMat, axis=0), 1)
    SLF = np.dot(self.U, self.V.T) # standard logistic function
    SLF += self.cell_biases
    SLF += self.locs_biases.T
    SLF = np.exp(SLF)
    SLF /= (SLF + self.ones)
    SLF = self.intMat1 * SLF
    if cell:
        vec_deriv -= np.dot(SLF, self.V)
        bias_deriv -= np.expand_dims(np.sum(SLF, axis=1), 1)
        vec_deriv -= self.lambda_p*self.U+self.alpha*np.dot(self.PL, self.U)
    else:
        vec_deriv -= np.dot(SLF.T, self.U)
        bias_deriv -= np.expand_dims(np.sum(SLF, axis=0), 1)
        vec_deriv -= self.lambda_l * self.V+self.beta*np.dot(self.DL, self.V)
```

```
        return (vec_deriv, bias_deriv)


    def log_likelihood(self):
        loglik = 0
        A = np.dot(self.U, self.V.T)
        A += self.cell_biases
        A += self.locs_biases.T
        B = A * self.intMat
        loglik += np.sum(B)
        A=np.array(A, dtype=np.float64)
        A = np.array(np.exp(A), dtype=np.float64)
        A += self.ones

        A = np.log(A)
        A = self.intMat1 * A
        loglik -= np.sum(A)

        loglik -= 0.5 * self.lambda_p * np.sum(np.square(self.U))+0.5 * self.lambda_l * np.sum(np.square(self.V))
        loglik -= 0.5 * self.alpha * np.sum(np.diag((np.dot(self.U.T, self.PL)).dot(self.U)))+0.5 * self.beta *
np.sum(np.diag((np.dot(self.V.T, self.DL)).dot(self.V)))
        return loglik


    def construct_neighborhood(self, cell_sim,cells_sim_2,drugMat):
        self.dsMat = drugMat - np.diag(np.diag(drugMat))
        self.PSMat = cell_sim - np.diag(np.diag(cell_sim))
        self.PSMat_2 = cells_sim_2 - np.diag(np.diag(cells_sim_2))
        if self.K1 > 0:
            S1 = self.get_nearest_neighbors(self.PSMat, self.K1)
            self.PL = self.laplacian_matrix(S1)
            S2 = self.get_nearest_neighbors(self.dsMat, self.K1)
            self.DL = self.laplacian_matrix(S2)
```

```
        S1_2 = self.get_nearest_neighbors(self.PSMat_2, self.K2)
        self.PL_2 = self.laplacian_matrix(S1_2)

    else:
        self.PL = self.laplacian_matrix(self.PSMat)
        self.DL = self.laplacian_matrix(self.dsMat)
        self.PL_2 = self.laplacian_matrix(self.PSMat_2)


def laplacian_matrix(self, S):
    x = np.sum(S, axis=0)
    y = np.sum(S, axis=1)
    L = 0.5*(np.diag(x+y) - (S+S.T))  # neighborhood regularization matrix
    return L

def get_nearest_neighbors(self, S, size):
    m, n = S.shape
    X = np.zeros((m, n))
    for i in xrange(m):
        ii = np.argsort(S[i, :])[::-1][:min(size, n)]
        X[i, ii] = S[i, ii]
    return X

def fix_model(self, W, intMat,drugMat, cell_sim,cells_sim_2, seed=None):



    self.cell_count, self.locs_count = intMat.shape
    self.ones = np.ones((self.cell_count, self.locs_count))
    self.intMat = self.c*intMat*W
    self.intMat1 = (self.c-1)*intMat * W + self.ones
```

```
        x, y = np.where(self.intMat > 0)
        self.train_cell, self.train_locs = set(x.tolist()), set(y.tolist())
        self.construct_neighborhood(cell_sim,cells_sim_2,drugMat)
        self.AGD_optimization(seed)



    def predict_scores(self, test_data):

        prot_ind = np.array(list(self.train_cell))
        train_cell_sim = self.PSMat[:, prot_ind]
        train_cell_sim_2 = self.PSMat_2[:, prot_ind]

        scores = []
        for p, l in test_data:

            ii = np.argsort(train_cell_sim_2[p, :])[::-1][:self.K2]

            val = np.sum(np.dot(train_cell_sim_2[p, ii], self.U[prot_ind[ii], :])*self.V[l, :])/np.sum(train_cell_sim_2[p, ii])
            val+=np.sum(np.dot(train_cell_sim_2[p, ii], self.cell_biases[prot_ind[ii]]))/np.sum(train_cell_sim_2[p, ii]) +
self.locs_biases[l]
            scores.append(np.exp(val)/(1+np.exp(val)))
        return np.array(scores)

    def predict_scores_2(self, test_data):

        lll_train=[]
        prot_ind = np.array(list(self.train_cell))
        train_cell_sim = self.PSMat[:, prot_ind]
        train_cell_sim_2 = self.PSMat_2[:, prot_ind]
        #scores = []
        Ic50_values=[]
```

```
        p_test=[]

        for p, l in test_data:
            ii = np.argsort(train_cell_sim_2[p, :])[::-1][:self.K2]
            val = np.sum(np.dot(train_cell_sim_2[p, ii], self.U[prot_ind[ii], :])*self.V[l, :])/np.sum(train_cell_sim_2[p, ii])
            val+=np.sum(np.dot(train_cell_sim_2[p, ii], self.cell_biases[prot_ind[ii]]))/np.sum(train_cell_sim_2[p, ii]) + 
self.locs_biases[l]
            Ic50_values.append(val)

        for i in range (len(test_data)):
            p_test.append(test_data[i])

        return  np.array(Ic50_values)

    def __str__(self):
        return "Model: NRLMF, c:%s, K1:%s, K2:%s, r:%s, lambda_p:%s, lambda_l:%s, alpha:%s, beta:%s,theta:%s, 
max_iter:%s" % (self.c, self.K1, self.K2, self.r, self.lambda_p, self.lambda_l, self.alpha, self.theta, self.max_iter)
```

function()

```
# -*- coding: utf-8 -*-
Created on  2018
@author: asus
"""

from __future__ import division
import os
import numpy as np
import pandas as pd
import scipy.stats
import math
```

```
from sklearn import metrics
from sklearn.metrics import confusion_matrix, precision_score, recall_score,roc_auc_score
from sklearn import metrics

def score_to_exact_rank(s):

    p=np.argsort(s)[::-1]

    return np.argsort(p)



def load_matrices(folder):
    with open(os.path.join(folder, "matrix of 0 and 1.txt"), "r") as raw:

        observation_mat = [line.strip("\n").split() for line in raw]

    with open(os.path.join(folder, "similarity matrix of drugs.txt"), "r") as raw:
        raw.next()

        drug_matt = [line.strip("\n").split()[1:] for line in raw]
    with open(os.path.join(folder, "Liu_similarity_For_drugs.txt"), "r") as raw:
        #raw.next()
        drug_matt_liu = [line.strip("\n").split()[0:] for line in raw]



    with open(os.path.join(folder, "Similarity matrix based on gene expression profile.txt"), "r") as raw:
        #raw.next()
        cell_sim_1 = [line.strip("\n").split()[0:] for line in raw]
```

```
with open(os.path.join(folder, "Similarity matrix based on copy number alteration.txt"), "r") as raw:
    #raw.next()
    cell_sim_2 = [line.strip("\n").split()[0:] for line in raw]

with open(os.path.join(folder, "Similarity matrix based on single nucloetid mutation.txt"), "r") as raw:
    #raw.next()
    cell_sim_3 = [line.strip("\n").split()[0:] for line in raw]



with open(os.path.join(folder, "Liu_similarity of IC50 values.txt"), "r") as raw:
    #raw.next()
    cell_sim_4 = [line.strip("\n").split()[0:] for line in raw]
with open(os.path.join(folder, "Matrix of k-nearest neighbr.txt"), "r") as raw:
    #raw.next()
    cell_sim_new = [line.strip("\n").split()[0:] for line in raw]



with open(os.path.join(folder, "matrix of IC50 values.txt"), "r") as raww:
    raww.next()
    print('****************************')
    observation_mat_IC50 = [line.strip("\n").split()[1:] for line in raww]



observation_mat = np.array(observation_mat, dtype=np.float64)
cell_sim_1 = np.array(cell_sim_1, dtype=np.float64)
drug_mat=np.array(drug_matt, dtype=np.float64)
drug_matt_liu=np.array(drug_matt_liu, dtype=np.float64)
cell_sim_2 = np.array(cell_sim_2, dtype=np.float64)
cell_sim_3 = np.array(cell_sim_3, dtype=np.float64)
cell_sim_4 = np.array(cell_sim_4, dtype=np.float64)
cell_sim_new=np.array(cell_sim_new, dtype=np.float64)
```

```
    #train:
    proteins_sim = ((1* cell_sim_1) + (5*cell_sim_4 )+(1* cell_sim_2)+(1* cell_sim_3)) / (8)  #compute weighted
average of similarities

    proteins_sim_2=cell_sim_new

    drug_mat_main=drug_mat

    return observation_mat, proteins_sim,proteins_sim_2,observation_mat_IC50,drug_mat_main
def apply_threshold(scores, thre):

    prediction = np.zeros_like(scores)
    for i in range(len(scores)):
        threshold = max(scores[i]) - ((max(scores[i]) - min(scores[i])) * (thre))
        for j in range(len(scores[i])):
            if (scores[i][j] <= threshold):
                prediction[i][j] = 0
            else:
                prediction[i][j] = 1
    return prediction

def apply_threshold_median(scores):
    count=0
    #print(len(scores[0]))  :98
   # print(len(scores))
    prediction = np.zeros_like(scores)
    for i in range(0, len(scores[0])):
        threshold = np.median ((scores[:,i]))
        print(threshold)
        for j in range(0, len(scores)):
```

```
            if scores[j][i] < threshold:
                prediction[j][i] = 0

            else:
                prediction[j][i] = 1

    # print(count)
    return prediction

def compute_evaluation_criteria_across_wholeMatrix(true_result, prediction,scores):
    pres = np.zeros(len(true_result))
    recs = np.zeros(len(true_result))

    ACC_new=0.0
    F1_score_new=0.0
    REC_new=0.0
    Specificity=0.0
    MCC_new=0.0

    AUC_new_new=0.0
    precision_new=0.0
    co=0.0
    tp_final=0
    tn_final=0
    fp_final=0
    fn_final=0
    fmeas=[]
    true_auc=[]
    score_auc=[]

    for i in range(len(true_result[0])):
```

```
    fpr, tpr, thresholds = metrics.roc_curve(true_result[:,i], scores[:,i], pos_label=1)


    if (math.isnan(metrics.auc(fpr, tpr))!=True):
        co=co+1
        AUC_new_new+= metrics.auc(fpr, tpr)

    #print('AUC_new_new',AUC_new_new)
    true_auc.append(true_result[:,i])
    score_auc.append(scores[:,i])

    returned= confusion_matrix(true_result[:,i], prediction[:,i]).ravel()
    if len(returned) == 4:
        tn, fp, fn, tp = returned

    if len(returned) == 1:
       tn= returned
       fp=0
       fn=0
       tp=0

    tp_final+=tp
    tn_final+=tn
    fp_final+=fp
    fn_final+=fn
#ACC = (tp_final / (tp_final + fp_final + fn_final))
ACC_new =((tp_final+tn_final)/ (tp_final + fp_final + fn_final+tn_final))
F1_score_new =((2*tp_final) / ((2*tp_final)+fp_final+ fn_final))
REC_new=((tp_final)/(tp_final+fn_final))
```

```
     # REC_new+=((tp)/(tp+fn))
    precision_new=((tp_final)/(tp_final+fp_final))

    MCC_new=(((tp_final*tn_final)-
(fp_final*fn_final))/(np.sqrt((tp_final+fp_final)*(tp_final+fn_final)*(fp_final+tn_final)*(fn_final+tn_final))))

    Specificity=((tn_final)/(tn_final+fp_final))

    AUC_new_new=AUC_new_new/(co)
    for i in range(0, len(true_result[0])):
      #  print('len(true_result)', len(true_result[0]))
        recall = 0
        precision = 0
        r_loc = 0
        p_loc = 0
        for j in range(0, len(true_result)):
            if true_result[j][i] == 1:
                recall += recs[j]
                r_loc += 1
            if prediction[j][i] == 1:
                precision += pres[j]
                p_loc += 1
        if r_loc != 0:
            recall = recall / r_loc
        else:
            recall = 0
        if p_loc != 0:
            precision = precision / p_loc
        else:
```

```
            precision = 0
        if (precision == 0 and recall == 0):
            fmeas.append(0)
        else:
            fmeas.append(round((2 * recall * precision) / (precision + recall), 4))
    finalF = np.mean(fmeas)
    return round(precision_new,2),round(ACC_new,2),round(F1_score_new,2),round(REC_new,2),round(Specificity,2),round(MCC_new,2),round(AUC_new_new,2), fmeas
```
